# Nonclinical Pharmacokinetics and Pharmacodynamics Characterization of Anti-CD79b/CD3 T Cell-Dependent Bispecific Antibody Using a Surrogate Molecule: A Potential Therapeutic Agent for B Cell Malignancies

**DOI:** 10.3390/pharmaceutics14050970

**Published:** 2022-04-30

**Authors:** Rajbharan Yadav, Siddharth Sukumaran, Tanja S. Zabka, Jinze Li, Amy Oldendorp, Gary Morrow, Arthur Reyes, Melissa Cheu, Jessica Li, Jeffrey J. Wallin, Siao Tsai, Laura Sun, Peiyin Wang, Diego Ellerman, Christoph Spiess, Andy Polson, Eric G. Stefanich, Amrita V. Kamath, Meric A. Ovacik

**Affiliations:** 1 Preclinical and Translational Pharmacokinetics and Pharmacodynamics, Genentech Inc., 1 DNA Way, South San Francisco, CA 94080, USA; ssid86@gmail.com (S.S.); reyes.arthur@gene.com (A.R.); erics@gene.com (E.G.S.); amritak@gene.com (A.V.K.); 2 Safety Assessment, Genentech Inc., 1 DNA Way, South San Francisco, CA 94080, USA; zabkat@gene.com (T.S.Z.); jinzeli@gmail.com (J.L.); arata@gene.com (A.O.); morrow.gary@gene.com (G.M.); 3 BioAnalytical Sciences, Genentech Inc., South San Francisco, CA 94080, USA; melissa@gene.com; 4 Oncology Biomarker Development (OBD), Genentech Inc., South San Francisco, CA 94080, USA; lijessica476@gmail.com (J.L.); wallinjj@yahoo.com (J.J.W.); 5 Biochemical and Cellular Pharmacology, Genentech Inc., 1 DNA Way, South San Francisco, CA 94080, USA; hsiaopingtsainpw20@gmail.com; 6 Translational Oncology Department, Genentech Inc., South San Francisco, CA 94080, USA; ls2020cal@gmail.com (L.S.); wangp33@gene.com (P.W.); polson@gene.com (A.P.); 7 Antibody Engineering, Genentech Inc., South San Francisco, CA 94080, USA; diegoe@gene.com (D.E.); christsp@gene.com (C.S.)

**Keywords:** T cell-dependent bispecific antibody, CD79b, CD3 binding affinity, pharmacokinetics, pharmacodynamics, target-mediated drug disposition model, internalization, surrogate molecule

## Abstract

The T cell-dependent bispecific (TDB) antibody, anti-CD79b/CD3, targets CD79b and CD3 cell-surface receptors expressed on B cells and T cells, respectively. Since the anti-CD79b arm of this TDB binds only to human CD79b, a surrogate TDB that binds to cynomolgus monkey CD79b (cyCD79b) was used for preclinical characterization. To evaluate the impact of CD3 binding affinity on the TDB pharmacokinetics (PK), we utilized non-tumor-targeting bispecific anti-gD/CD3 antibodies composed of a low/high CD3 affinity arm along with a monospecific anti-gD arm as controls in monkeys and mice. An integrated PKPD model was developed to characterize PK and pharmacodynamics (PD). This study revealed the impact of CD3 binding affinity on anti-cyCD79b/CD3 PK. The surrogate anti-cyCD79b/CD3 TDB was highly effective in killing CD79b-expressing B cells and exhibited nonlinear PK in monkeys, consistent with target-mediated clearance. A dose-dependent decrease in B cell counts in peripheral blood was observed, as expected. Modeling indicated that anti-cyCD79b/CD3 TDB’s rapid and target-mediated clearance may be attributed to faster internalization of CD79b, in addition to enhanced CD3 binding. The model yielded unbiased and precise curve fits. These findings highlight the complex interaction between TDBs and their targets and may be applicable to the development of other biotherapeutics.

## 1. Introduction

B cell lymphomas are clonal tumors of mature and immature CD20+ B lymphocytes (B cells) that constitute the majority (80–85%) of non-Hodgkin lymphomas (NHLs) and other leukemias, such as acute lymphoblastic leukemia (ALL) and chronic lymphocytic leukemia (CLL). Among the emerging molecules for the treatment of hematologic malignancies, bispecific T cell-dependent antibodies (TDB), which rely on immune-mediated mechanisms, represent a promising approach [1]. T cell-dependent bispecific antibodies typically bind to both CD3ε, a subunit of the T cell receptor complex, and a target antigen expressed on cancer cells in order to recruit and redirect patient T lymphocytes to tumor cells for elimination [2,3]. Each arm of the bispecific antibody can form a binary complex after binding to its specific target (i.e., CD3 and the target antigen). The binary complexes can further bind to the other target to form the ternary complex or synapse [4]. This ternary complex is the pharmacologically active species that drives the pharmacodynamics (PD) of the bispecific antibody (e.g., designed to redirect T cells toward tumor cells). Thus, ternary complex concentration is relevant for elucidating the PD behavior of bispecific antibodies [5,6,7].

While bispecific antibodies are not new [8], improvements in engineering have renewed interest in this antibody construct, and there are multiple promising drugs currently in clinical development [9,10,11]. In this regard, the anti-CD3/CD19 bispecific T cell engager, blinatumomab, a fusion protein of two single-chain Fv antibody fragments, received FDA approval in 2014 for the treatment of a rare form of ALL [12,13], and resulted in complete responses in 26 of 36 (72%) ALL patients [14]. Mosunetuzumab, a humanized full-length anti-CD20/CD3 T cell-dependent bispecific antibody, with a similar mechanism of action to that of blinatumomab, demonstrated that concurrent binding to CD20 on malignant B cells and CD3 on T cells leads to T cell activation and B cell lysis [15,16].

CD79b, a component of the B cell receptor complex, was clinically validated as a safe and effective therapeutic target for B cell malignancies using an anti-CD79b antibody-drug conjugate (ADC) [17]. We have demonstrated that an anti-CD79b/CD3 TDB is active against lymphoma cells, with a wide range of CD79b levels in vitro. In addition, anti-CD79b/CD3 TDB administration inhibited tumor growth in B cell lymphoma xenograft models and resulted in potent B cell depletion in the blood and spleens in a humanized murine model of lymphoma [18].

A challenge for the preclinical pharmacokinetics (PK), PD, and safety assessment of anti-CD79b/CD3 TDB (clinical candidate) was that this TDB only binds to human CD79b resulting in the lack of a pharmacologically relevant nonclinical species with which to evaluate antigen-dependent PK, pharmacology, and safety, prior to first-in-human clinical trials (ICH S6, 2011 and ICH S9, 2009). In such cases, alternative approaches to the nonclinical assessment of antibody-based therapeutics can include the use of surrogate molecules, surrogate animal models, or in vitro pharmacological systems (ICH S6, 2011 and ICH S6 addendum, 2012). Thus, in order to assess the pharmacological effect of clinical candidate anti-CD79b/CD3 TDB in non-human primates, we generated a surrogate anti-cyCD79b/CD3 TDB, with comparable in vitro potency to the anti-human CD79b-expressing TDB antibody. This surrogate TDB has a target arm that recognizes cynomolgus monkey CD79b and expresses the same anti-CD3 arm as the human TDB antibody, which cross-reacts with cynomolgus CD3. We also produced anti-gD/CD3 TDB antibodies with low or with high CD3 binding affinities, to determine the effect of CD3 binding alone on PKPD and to inform understanding of the PKPD of the anti-CD79b/CD3 TDB. Anti-gD is a non-targeted antibody which was used to produce anti-gD/CD3 TDB antibodies. Notably, all TDB antibodies were generated using knob-into-hole (KIH) mutations [19,20,21].

The development of a mechanistic PKPD model for TDBs is complicated, due to the differences in kinetics and binding affinities of both targets. Numerous models have been developed to describe the PK and PD biomarker of TDBs that bind to soluble or membrane bound targets by incorporating target dynamics (e.g., baseline levels and turnover rates), affinity, and avidity parameters and have been used to characterize PK, PD, and safety of TDBs [4,5,16,22,23]. However, all these models use parameters that are difficult to accurately measure, for example, the rapid processes such as binding reactions and internalization of the complexes. Thus, there is a need for a reliable approximation of a generic TDB model, which can characterize and predict the PK and PD with fewer parameters. Here, we have presented a full bispecific TMDD model that is applied to investigate the PK behavior of the TDBs and predict ternary complex concentrations, which was used to drive the PD biomarker changes.

For this study, we systematically characterized the PK and the changes in B cell numbers as the PD marker of anti-CD79b/CD3 clinical candidate efficacy in cynomolgus monkeys using the surrogate anti-cyCD79b/CD3 TDB molecule. We also determined the effect of CD3 binding affinity on PKPD using anti-gD/CD3 TDBs with a low or a high affinity CD3 arm. Finally, we developed an integrated PKPD model to characterize the PK and PD of the surrogate anti-cyCD79b/CD3 TDB in cynomolgus monkeys and help to translate clinical candidate TDB into patients.

## 2. Materials and Methods

### 2.1. Generation of Test Materials

We adopted a KIH antibody format and produced TDBs as full length, humanized IgG1 antibodies with natural antibody architecture [15,24], which allows one arm to target CD79b while the other arm recruits T cells by binding to the CD3ε subunit of the T cell receptor. The surrogate (anti-cyCD79b/CD3) and anti-CD79b/CD3 TDBs bind to the same N-terminal region on the corresponding CD79b s and express the same high affinity CD3 arm. Bispecific controls, anti-gD/CD3 TDBs, with a low or with a high affinity CD3 arm (denoted anti-gD/CD3-low affinity and anti-gD/CD3-high affinity), were generated to test the impact of CD3 binding on PK and PD [25]. An antibody (anti-gD) to HSV-1 viral coat protein gD (humanized IgG1 5B6) was used as a non-targeted control antibody [26]. Anti-gD was produced and humanized at Genentech, Inc. (South San Francisco, CA, USA) [26].

### 2.2. Human and Cyno CD79b Affinity Meaurement

The binding affinity (K_D_) of the anti-CD79b/CD3 and anti-cyCD79b/CD3 TDB was determined by surface plasmon resonance (SPR) using a Biacore T200 instrument (GE Healthcare, Chicago, IL, USA). For kinetics measurements, CD79b protein was coupled to Biacore research-grade CM5 chips, in accordance with the instructions of the supplier, to achieve approximately 100 response units in each flow cell. Purified anti-CD79b/CD3 and anti-cyCD79b/CD3 antibodies or Fab variant (a 2-fold serial dilution of 0.5 to 1000 nM in PBST) were injected at a flow rate of 30 µL/min. Each sample was analyzed with 4 min association and 10 min disassociation. After each injection the chip was regenerated using 10 mM glycine pH 1.7. Binding response was corrected by subtracting a control flow cell from anti-CD79b variant IgG flow cells. All experiments were conducted at 25 °C. Association rates (ka) and dissociation rates (kd) were calculated using a 1:1 Langmuir binding model (Biacore T200 Evaluation Software version 2.0, Chicago, IL, USA). The equilibrium dissociation constant (K_D_) was calculated as the ratio kd/ka.

### 2.3. Human and Cyno CD3ε Affinity Measurement

Human and cynomolgus monkey CD3ε peptides (Genentech) were used to determine the K_D_ of anti-CD79b/CD3 TDBs for CD3ε by SPR using a Biacore T200 instrument (GE Healthcare, Chicago, IL, USA) as described previously [25]. In brief, biotinylated peptides were immobilized to FC2, FC3, and FC4 of a Series S Sensor Chip Streptavidin (GE Healthcare). No peptide was immobilized to FC1, which was used as an in-line reference for FC2, FC3, and FC4. Various concentrations of anti-CD79b/CD3 were then injected into all FCs for 5 min, and dissociation was allowed to proceed for 2 min. At the end of each association/dissociation cycle, the chip was regenerated using 3M MgCl2. All experiments were conducted at 37 °C. The off-rate for the low-affinity CD3 molecule was too fast to obtain kinetic information. Therefore, steady-state binding analysis was used to derive CD3 K_D_ for all anti-CD79b/CD3 molecules using Biacore Evaluation Software (Chicago, IL, USA).

The CD3 arms of anti-CD79b/CD3 TDBs are cross-reactive with cyno and human CDε and not cross-reactive to mouse CD3ε. These TDBs binds to the N-terminus of CD3ε in cyno and humans, and there is significant sequence divergence between rodents (mice and rats) and humans [27]. Furthermore, an alignment of CD3ε across several species demonstrates general lack of sequence identity outside of the primate family [28,29].

### 2.4. Ethics Statement

The authors confirm that they have obtained appropriate institutional review board approval for all animal experimental investigations at both Genentech and Charles River Laboratories (Reno, NV, USA). All procedures were approved by the Institutional Animal Care and Use Committee (IACUC) at Genentech and at Charles River Laboratories and were performed in compliance with the Animal Welfare Act, the Guide for the Care and Use of Laboratory Animals, and the Office of Laboratory Animal Welfare. The in vivo PK studies in SCID.beige (SCID.bg) mice were conducted at Genentech, Inc., and all the single and repeat-dose pharmacokinetics/toxicokinetics studies in cynomolgus monkeys were conducted at Charles River Laboratories. The four-week repeat-dose study was performed in accordance with the United States Food and Drug Administration (United States Code of Federal Regulations, Title 21, Part 58) Good Laboratory Practice (GLP) regulations for Nonclinical Laboratory Studies and as accepted by regulatory authorities throughout the European Union (Organization for Economic Co-operation and Development (OECD) Principles of Good Laboratory Practice) and Japan (Ministry of Health, Labour and Welfare, Tokyo, Japan). The toxicokinetic analysis of this study conducted at Charles River Laboratories in Canada (Montreal, QC, Canada, ULC) was performed in accordance with the OECD Principles of Good Laboratory Practice as accepted by regulatory authorities throughout the European Union, United States of America, and Japan.

### 2.5. In Vitro B Cell Killing

B cell killing by the surrogate (anti-cyCD79b/CD3) or the clinical candidate (anti-CD79b/CD3) TDBs was determined using transfected BJAB (BJAB.PD.cyCD79b.E3) cells, a Burkitt’s lymphoma-derived B cell line that expresses both human and cynomolgus monkey CD79b [30]. In brief, CD8+ T effectors cells isolated from two healthy donors and the BJAB target cells were incubated for 24 h, with a range of concentrations of anti-cyCD79b/CD3 or anti-CD79b/CD3 TDBs with an effector to target cell ratio of 5:1 (E:T = 5:1). At the end of the incubation, the number of live B cells were counted by gating on CD19+/PI-population by FACS, and absolute cell count was obtained with fluorescein isothiocyanate beads added to the reaction mix as an internal counting control. The percent of B cell killing was calculated as follows: (live B cell number without TDB-live B cell number with TDB)/(live B cell number without TDB) × 100. BJAB.PD.cyCD79b.E3 cells were obtained from the Genentech (RRID: SCR_003997) cell line repository and maintained in RPMI-1640 supplemented with 10% FBS (Sigma) and 2 mM L-glutamine.

### 2.6. In Vivo PK Study in SCID.Beige Mice

Female SCID.bg mice received a single IV dose of 3 mg/kg of clinical candidate (anti-CD79b/CD3), cynomolgus surrogate (anti-cyCD79b/CD3) TDB, or three control antibodies: anti-gD (monospecific, bivalent) and two anti-gD/CD3 TDBs (bispecific) with a low or with a high affinity CD3 arm (denoted as anti-gD/CD3-low affinity and anti-gD/CD3-high affinity), via the tail vein (sparse sampling approach; n = 9 mice per group; n = 3 mice/timepoints). Blood samples were collected via retro-orbital bleeds conducted on alternate eyes, and the terminal blood sample was collected via cardiac puncture. Three blood samples were taken from each mouse; there were three mice per time point. Samples were collected at the following time points: pre-dose, 15 min, 6 and 24 h, and 2, 3, 7, 10, 14, and 21 days post-dose, and processed to collect serum. Group mean serum concentration-time profiles were constructed to estimate relevant PK parameters.

### 2.7. Single-Dose PK of Anti-CD79b/CD3 TDB, and Anti-gD/CD3-Low or -High Affinity TDBs in Cynomolgus Monkeys

A total of nine male cynomolgus monkeys were assigned to three treatment groups (n = 3 animals/group). Monkeys in each group received a single IV dose of 1 mg/kg of anti-CD79b/CD3 (clinical candidate), anti-gD/CD3-low affinity, or anti-gD/CD3-high affinity TDBs. Blood samples were collected at following time points for PK measurements: pre-dose, 5 min, 2 and 6 h post-dose, and 1, 2, 4, 7, 10, 14, 21, 28, and 34 days post-dose. Anti-drug antibodies (ADA) samples were collected at following time points: pre-dose (7 days prior to dosing), and 14, 21, 28, and 34 days post-dose. PK of anti-gD was characterized in another study at 3 mg/kg, following IV administration in a cynomolgus monkey. The dose-normalized PK profile and parameters at 1 mg/kg are reported in the Section 3 below.

### 2.8. Dose Ranging PKPD of the Surrogate Anti-cyCD79b/CD3 TDB in Cynomolgus Monkeys

Anti-cyCD79b/CD3 TDB is a cynomolgus monkey surrogate of the human anti-CD79b/CD3 TDB antibody. Male (n = 4/dose group) cynomolgus monkeys were assigned to each of three treatment groups and given a single IV dose of 0.01, 0.1, or 0.3 mg/kg of anti-cyCD79b/CD3 TDB each to evaluate the PK over a 30-fold dose range. Blood samples (1 mL) for PK and ADA analyses were collected from each animal via the femoral vein and processed for serum. PK samples were collected on day 0 (pre-dose), 5 min, 2 and 6 h post-dose, and 1, 2, 4, 7, 10, 14, 21, and 29 days post-dose, while ADA samples were collected pre-dose (7 days prior to dosing), and 14, 21, and 29 days post-dose. ADA samples were not analyzed. Blood samples were collected at designated time points to evaluate the dose-dependent effect of TDB treatment on CD20+ B lymphocytes.

### 2.9. Single- or Multiple-Dose Toxicokinetics (TK) of the Surrogate Anti-cyCD79b/CD3 TDB via IV or Sub-Cutaneous (SC) Administration in Cynomolgus Monkeys

Nine male cynomolgus monkeys were randomly assigned to three groups (n = 3 animals/group) and were given a single IV dose of vehicle (Group 1) or anti-cyCD79b/CD3 TDB (Groups 2 and 3). Animals in Group 2 received a 1 mg/kg, single IV dose of anti-cyCD79b/CD3 TDB, while animals in Group 3 received three daily 1.5 mg/kg/dose SC doses of anti-cyCD79b/CD3 TDB. At selected time points up to 7 days post-dose, serum samples were collected and analyzed for anti-cyCD79b/CD3 serum concentrations using ELISA. Serum concentration-time data were used to evaluate TK. Blood samples were collected at designated time points for flow cytometric evaluation of CD20+ B lymphocytes.

### 2.10. 4-Week Repeat-Dose GLP Study of Anti-cyCD79b/CD3 TDB Administered via IV Infusion in Cynomolgus Monkeys with a 7-Week Recovery Period

Naïve male and female cynomolgus monkeys (n = 5 animals/sex/group) were given an IV infusion (~1 h) of vehicle and 1 mg/kg of anti-cyCD79b/CD3 TDB, once every week (QW) for a total of four dose cycles (on days 0, 7, 14, and 21). Blood samples (1 mL) for PK and ADA analyses were collected from each animal via the femoral vein and processed for serum. The PK of anti-cyCD79b/CD3 TDB was quantified pre-dose, 15 min, 2 and 6 h, and 1, 3, 7 days after the first and fourth (last) QW doses, as well as pre-dose and 0.25 h after the second and third QW doses. PK samples from recovery animals (n = 4 (2F/2M) of a total of 10 animals) were collected for 7 weeks (on days 34, 49, 63, and 78) after the fourth dose of anti-cyCD79b/CD3 TDB. ADA samples were collected pre-dose (two weeks prior to dosing), and at days 7, 14, 28, 49, and 78 post-dosing. Blood samples were collected at designated time points for flow cytometric evaluation of CD20+ B lymphocytes.

### 2.11. Peripheral Blood Immunophenotyping

Blood samples (∼0.6 mL) were collected throughout the study for peripheral blood immunophenotyping by flow cytometric analysis for B lymphocytes (CD4−/CD8−/CD20+). The relative percentages of each phenotype obtained from the flow cytometer were multiplied by the absolute lymphocyte count from the hematology analysis in order to enumerate absolute cell counts.

### 2.12. PK/TK Assay Evaluation in Mice and in Cynomolgus Monkeys

Serum samples from SCID.bg mice and from cynomolgus monkeys were analyzed for anti-CD79b/CD3, anti-cyCD79b/CD3, anti-gD/CD3-low affinity, and anti-gD/CD3-high affinity TDBs, and anti-gD concentrations using a quantitative ELISA. Briefly, this ELISA uses a sheep anti-human immunoglobulin (IgG) heavy and light chain antibody as the capturing reagent and goat anti-human IgG heavy and light chain conjugated to horseradish peroxidase (HRP) (Cat# 200–032-156, Jackson ImmunoResearch, West Grove, PA, USA) as the detecting reagent. The lower limit of quantitation (LLOQ) was 1.56 ng/mL, for both SCID.bg mice and cynomolgus serum assays.

The concentration of anti-cyCD79b/CD3 in repeat-dose GLP TK serum samples was determined by PPD^®^ Laboratories (Richmond, VA, USA), using a validated liquid chromatography with tandem mass spectrometry (LC-MS/MS) method in cynomolgus monkey serum. An affinity capture approach using streptavidin magnetic beads coupled to biotinylated mouse anti-human IgG antibody was used to enrich anti-cyCD79b/CD3 (IgG1) and internal standard, SILuMab, from cynomolgus monkey serum. The bound proteins were subjected to “on-bead” proteolysis with trypsin, following standard protein denaturation, reduction, and alkylation processing steps. The characteristic peptide fragments produced by this procedure were then quantified as surrogates of the total antibody concentration originating from anti-cyCD79b/CD3 by LC/MS/MS (i.e., multiple reaction monitoring or MRM). The LLOQ for neat samples was 0.0500 μg/mL.

### 2.13. Anti-Drug Antibody (ADA) Assay

Cynomolgus monkey serum samples were analyzed by a generic immunocomplex ADA immunoassay to detect ADAs against anti-CD79b/CD3, anti-gD/CD3-low affinity, or anti-gD/CD3-high affinity TDBs [31,32,33]. The assay cut point was determined from a panel of therapeutic-naïve individual serum samples. ADAs against anti-cyCD79b/CD3 were determined by PPD^®^ Laboratories (Richmond, VA, USA), using a validated ELISA-based method. The detection of ADAs to anti-cyCD79b/CD3 were performed using a bridging ELISA assay that utilized a combination of biotin-conjugated anti-cyCD79b/CD3 and digoxin-conjugated anti-cyCD79b/CD3 to capture ADAs, along with mouse anti-digoxin antibody conjugated with HRP (Cat# 200–032-156, Jackson ImmunoResearch, West Grove, PA, USA). for detection. Using a mouse anti-human IgG antibody as a surrogate positive control, the relative sensitivity was determined to be 75 ng/mL, and the assay was able to detect 1000 ng/mL positive control source material in the presence of up to 10.0 μg/mL free anti-cyCD79b/CD3 TDB.

### 2.14. PK/TK Data Analysis

Serum-concentration-time profiles were used to estimate the PK parameters in mouse and monkey, using non-compartmental analysis (NCA) (Phoenix™ WinNonlin^®^, Version 6.4; Pharsight Corporation; Mountain View, CA, USA). For SCID mouse, the plasma concentration vs. time data were naïve pooled together (sparse sampling approach) to provide one PK parameter estimate for each dose group, while in monkey, each animal was analyzed separately, and results for each dose group were summarized as mean ± standard deviation (SD). Therefore, SD is not provided for all PK parameters, and the reported mean parameter represents composite value from naïve pooling of animals for SCID mouse study. The reported parameter variability in C_max_ from SCID mice (since observed at first time point (at 15 min) from n = 3 mice) represents standard error of mean (SEM) and is the result of a naïve pool approach with non-compartmental analysis. AUC was calculated using the log-linear trapezoidal rule. Nominal sample collection times and nominal dose solution concentrations were used in data analysis. For the single-dose studies in mice and monkeys, C_max_, area under the serum concentration-time curve from the end of infusion to the last measurable time point (AUC_0–last_), area under the serum concentration-time curve extrapolated to infinity (AUC_0–∞_), systemic clearance (CL), and volume of distribution at steady state (V_ss_) were calculated. Additionally, T_max_ (Time of observed C_max_) was reported for treatment group given three daily SC doses of anti-cyCD79b/CD3 TDB. For the repeat-dose cynomolgus study, C_max_ after first drug administration (TK day 0) and fourth drug administration (TK day 21), AUC in the first dosing interval (AUC_0–7_) and fourth dosing interval (AUC_21–28_), and from the end of infusion to the last measurable time point (AUC_0–last_) were obtained. Descriptive statistics (means and standard deviations) and accumulation ratios (AUC_21–28_/AUC_0–7_) for male and females were generated. No animals were excluded from the statistical analysis.

### 2.15. Bispecific TMDD Model Development

**Figure 1:**
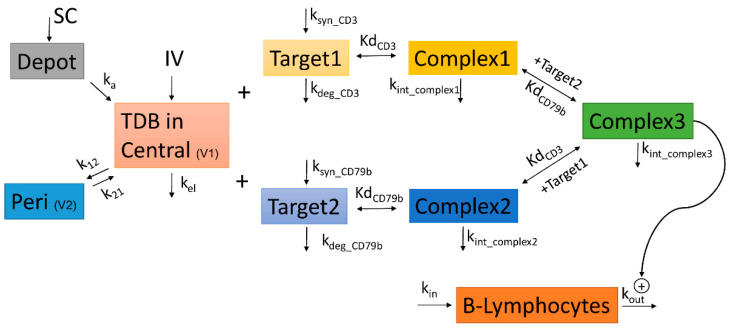
Model schematic to the described PKPD relationship in cynomolgus monkeys. The systemic PK of surrogate (anti-cyCD79b/CD3) TDB was described using a bispecific TMDD model, where anti-cyCD79b/CD3 TDB in systemic (C_BsAb_, V1) can distribute to peripheral tissue (k_12_, k_21_, V2), be eliminated (kel = CL/V1), and bind to CD3 or CD79b (k_on_CD3_ or _CD79b_, Kd__CD3_ or _CD79b_) to form two binary complexes; both of these binary complexes further binds to other target to form ternary complex [4,7]. The baseline level of CD3 and CD79b were expressed as k_syn_CD3_ or _CD79b_/k_deg_CD3_ or _CD79b_. The drug effect is modeled by the indirect response model; the ternary complex concentration is linked to promotion on B cells (CD20+ B lymphocytes) elimination via a stimulation function (plus sign indicate E = (Emax × Complex3)/(EC50 + Complex3) [37]. Detailed explanation and model estimate of the parameters are shown in Table 6.

A bispecific target-mediated drug disposition (TMDD) model was established to characterize the pharmacokinetics of anti-cyCD79b/CD3 TDB concentrations, following single or repeat dose IV or SC administrations in cynomolgus monkeys with quasi-equilibrium assumption [5,7,34,35,36]. To model the binding kinetics of the bispecific (BsAb), we assume that free BsAb binds to either of two targets, CD3 or CD79b, to form binary complexes: Complex1 or Complex2, respectively [4]. Binary complexes can subsequently bind to the other target to form the ternary complex, Complex3. All reactions are assumed to follow first-order elementary reversible reaction kinetics. We assumed that the already-bound (first) arm would not interfere with nor facilitate the BsAb binding to the other arm. The model consists of four binding events between six species, leading to the formation of binary and ternary complexes. Targets (CD3 and CD79b) are generated using zero-order rates k_syn_CD3_ or k_syn_CD79b_ and degraded at first-order rates k_deg_CD3_ or k_deg_CD79b_. All three complexes are assumed to internalize with rates k_int_Complex1_, k_int_Complex2_, or k_int_Complex3_. The drug is assumed to eliminate linearly from the central compartment with k_el_ and distributes to the peripheral compartment via k_12_ and k_21_. For consistency in units of parameter estimates, all concentration data with microgram per milliliter units (µg/mL) were converted to equivalent nanomolar concentrations (nM), and doses were converted to nanomole/kg by using the molecular weight of 150 kDa of a typical antibody. The model structure is presented in Figure 1, and the equations that describe the model are shown below:

(1) $$\begin{array}{cc} \frac{{\mathrm{dC}}_{\mathrm{BsAb}\;}}{\mathrm{dt}}{=k}_{\mathrm{el}}{\cdot C}_{\mathrm{BsAb}} & -{\;k}_{\mathrm{on}\_\mathrm{CD}3}\cdot C_{\mathrm{BsAb}}{\cdot C}_{\mathrm{Target}1}{+(k}_{\mathrm{on}\_\mathrm{CD}3}{\cdot k}_{D\_\mathrm{CD}3})\cdot \mathrm{Complex}1\;\\ & -{\;k}_{\mathrm{on}\_\mathrm{CD}79b}\cdot C_{\mathrm{BsAb}}{\cdot C}_{\mathrm{Target}2}{+(k}_{\mathrm{on}\_\mathrm{CD}79b}{\cdot k}_{D\_\mathrm{CD}79b})\cdot \mathrm{Complex}2\;\\ & -{\;k}_{12}{\cdot C}_{\mathrm{BsAb}}{+k}_{21}\cdot \frac{A\_\mathrm{Peri}}{V_{1}}{+k}_{a}\cdot \frac{\mathrm{SC}}{V_{1}}\; \end{array}$$

(2) $$\begin{array}{cc} \frac{{\mathrm{dC}}_{\mathrm{Target}1}}{\mathrm{dt}} & =k_{\mathrm{synCD}3}-k_{\deg\_\mathrm{CD}3}\cdot C_{\mathrm{Target}1}-{\;k}_{\mathrm{on}\_\mathrm{CD}3}\\ & \cdot C_{\mathrm{Target}1}\cdot C_{\mathrm{BsAb}}{+(k}_{\mathrm{on}\_\mathrm{CD}3}\cdot k_{D\_\mathrm{CD}3})\cdot \mathrm{Complex}1\\ & -k_{\mathrm{on}\_\mathrm{CD}3}\cdot \mathrm{Complex}2\cdot C_{\mathrm{Target}1}+\\ & {(k}_{\mathrm{on}\_\mathrm{CD}3}\cdot k_{D\_\mathrm{CD}3})\cdot \mathrm{Complex}3\; \end{array}$$

(3) $$\begin{array}{cc} \frac{{\mathrm{dC}}_{\mathrm{Target}2}}{\mathrm{dt}} & =k_{\mathrm{synCD}79b}-k_{\deg\_\mathrm{CD}79b}\cdot C_{\mathrm{Target}2}-{\;k}_{{\mathrm{on}}_{\mathrm{CD}79b}}\\ & \cdot C_{\mathrm{Target}2}\cdot C_{\mathrm{BsAb}}{+(k}_{\mathrm{on}\_\mathrm{CD}79b}\cdot k_{D\_\mathrm{CD}79b})\cdot \mathrm{Complex}2\;\\ & -{\;k}_{\mathrm{on}\_\mathrm{CD}79b}\cdot \mathrm{Complex}1\cdot C_{\mathrm{Target}2}\;{+(k}_{\mathrm{on}\_\mathrm{CD}79b}\cdot k_{D\_\mathrm{CD}79b})\cdot \mathrm{Complex}3 \end{array}$$

(4) $$\begin{array}{cc} \frac{\mathrm{dComplex}1}{\mathrm{dt}} & ={\;k}_{\mathrm{on}\_\mathrm{CD}3}\cdot C_{\mathrm{Target}1}\cdot C_{\mathrm{BsAb}}-{\;(k}_{\mathrm{on}\_\mathrm{CD}3}\cdot k_{D\_\mathrm{CD}3})\cdot \mathrm{Complex}1-{\;k}_{\mathrm{int}\_\mathrm{Complex}1}\\ & \cdot \mathrm{Complex}1\\ & -k_{\mathrm{on}\_\mathrm{CD}79b}\cdot \mathrm{Complex}1\cdot C_{\mathrm{Target}2\;}{+(k}_{\mathrm{on}\_\mathrm{CD}79b}\cdot k_{D\_\mathrm{CD}79b})\cdot \mathrm{Complex}3 \end{array}$$

(5) $$\begin{array}{cc} \frac{\mathrm{dComplex}2}{\mathrm{dt}} & ={\;k}_{\mathrm{on}\_\mathrm{CD}79b}\cdot C_{\mathrm{Target}2}\cdot C_{\mathrm{BsAb}}-{\;(k}_{\mathrm{on}\_\mathrm{CD}79b}\cdot k_{D\_\mathrm{CD}79b})\cdot \mathrm{Complex}2\\ & -{\;k}_{\mathrm{int}\_\mathrm{Complex}2}\cdot \mathrm{Complex}2\\ & -k_{\mathrm{on}\_\mathrm{CD}3}\cdot \mathrm{Complex}2\cdot C_{\mathrm{Target}1\;}{+(k}_{\mathrm{on}\_\mathrm{CD}3}\cdot k_{D\_\mathrm{CD}3})\cdot \mathrm{Complex}3\; \end{array}$$

(6) $$\begin{array}{c} \frac{\mathrm{dComplex}3}{\mathrm{dt}}\\ ={\;k}_{\mathrm{on}\_\mathrm{CD}3}\cdot \mathrm{Complex}2\cdot C_{\mathrm{Target}1\;}{+k}_{\mathrm{on}\_\mathrm{CD}79b}\cdot \mathrm{Complex}1\cdot C_{\mathrm{Target}2\;}{+[((k}_{\mathrm{on}\_\mathrm{CD}79b}\cdot k_{D\_\mathrm{CD}79b}{)+(k}_{\mathrm{on}\_\mathrm{CD}3}\cdot k_{D\_\mathrm{CD}3})+\\ {(k}_{\mathrm{int}\_\mathrm{Complex}3}))]\cdot \mathrm{Complex}3\; \end{array}$$

(7) $$\frac{{\mathrm{dA}}_{\mathrm{Peri}}}{\mathrm{dt}}{=k}_{12}\cdot C_{\mathrm{BsAb}}\cdot V1-{\;k}_{12}\cdot A_{\mathrm{Peri}}$$

(8) $$\frac{\mathrm{dSC}}{\mathrm{dt}}=-{\;k}_{a}\cdot \mathrm{SC}$$

(9) $$\mathrm{SC}\;(0)=\frac{F*\mathrm{Dosenmole}}{V_{1}}$$

(10) $$C_{\mathrm{BsAb}}=\frac{\mathrm{Dosenmole}}{V_{1}}$$

The B cell counts following IV and SC administration of anti-cyCD79b/CD3 TDB in the three studies described above were also used to develop the PD model (Figure 1). A turnover model was developed to describe the life cycle of CD20+ B lymphocytes, including the effect of TDB on increasing death rate of CD20+ B cells [37]. Specifically, following anti-cyCD79b/CD3 TDB treatment, formation of ternary complex (concentration) was assumed to drive peripheral CD20+ B lymphocyte depletion via stimulation of loss of B cells (R), which represents the response biomarker [5,7]. Peripheral B cell depletion was described by an indirect response model integrated with the bispecific TMDD model for the anti-cyCD79b/CD3 TDB described above. Complex-induced stimulation of B cell loss is modeled with an E_max_ and EC_50_, as described in Equation (11), which described the PD model:

(11) $$\frac{\mathrm{dR}}{\mathrm{dt}}=k_{\mathrm{in}}-k_{\mathrm{out}}\cdot \;[1+\frac{E_{\max}\cdot \mathrm{Complex}3}{{\mathrm{EC}}_{50}+\mathrm{Complex}3}]\cdot R$$

In the above equation, R represents the B cell count, k_in_ is the apparent zero-order rate constant for production of CD20+ B lymphocytes, k_out_ is the first-order rate constant for elimination of CD20+ B lymphocytes, E_max_ is the maximum increase in cell elimination rate in the presence of TDB (ternary complex), and the EC_50_ is the ternary complex concentration that produces 50% of maximum stimulation achieved. In the absence of TDB at steady-state, the baseline B cell count is given by k_in_/k_out_ and we estimated baseline and fixed B cells turnover (k_out_) at a value of 0.0126 (1/day) [38], which corresponds to a half-life of 55 days [38]. First, the PK data were used to fit the bispecific TMDD model. Subsequently, the bispecific TMDD model and the estimated parameters were used to fit the CD20+ B lymphocyte count data to the PD model.

### 2.16. Estimation

All model parameters were estimated with the Stochastic Approximation Expectation Maximization (SAEM) algorithm in Monolix (version 2020R1; http://www.lixoft.com/). Each model parameter is defined by a typical value and by its variability within the subject population. Simulations were performed by Simulx 2020R1.

## 3. Results

### 3.1. In Vitro Binding Affinity of TDBs

Table 1 shows the binding affinities of each arm of the TDB antibodies used in the cynomolgus monkey study. Both clinical candidate (anti-CD79b/CD3) and surrogate (anti-cyCD79b/CD3) TDB contain the same high affinity (14.4 nM to human CD79b and 12.8 nM to cynomolgus CD3) CD3 arm. The CD79b and cyCD79b arm of each TDB antibody binds to the same N-terminal region on its target receptor (CD79b), with binding affinities of 17 nM (clinical candidate) and 1 nM (cynomolgus surrogate), respectively. Control bispecific anti-gD/CD3 TDB with a low (387 nM; cynomolgus CD3) or a high (12.8 nM; cynomolgus CD3) affinity CD3 arm (denoted as anti-gD/CD3-low affinity and anti-gD/CD3-high affinity, respectively) were included in the study to evaluate the impact of CD3 binding affinity on PKPD and are cross-reactive with cynomolgus CD3ε.

### 3.2. Anti-CD79b/CD3 and Cynomolgus Surrogate Anti-cyCD79b/CD3 TDBs Are Active against Human and Cynomolgus Monkey CD79b-Expressing B Lymphoma (BJAB) Cell Lines

**Figure 2:**
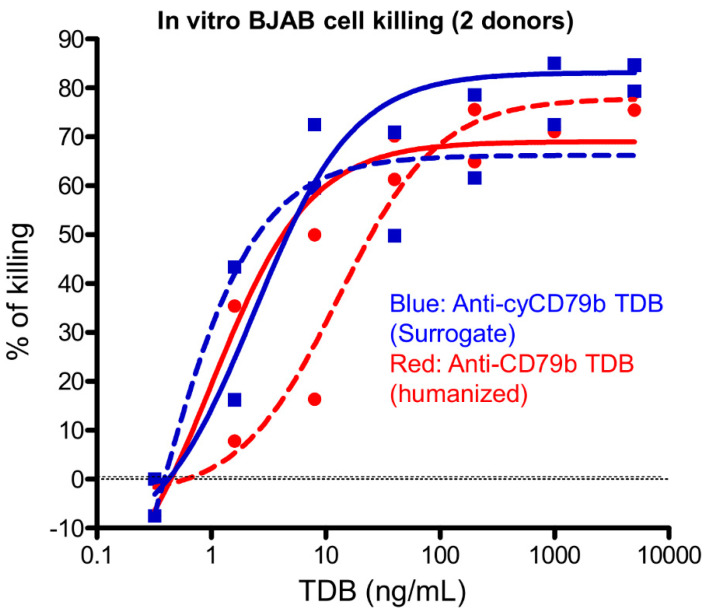
Surrogate (anti-cyCD79b/CD3) and clinical candidate (anti-CD79b/CD3) TDBs are potent in killing cynomolgus lymphoma B cell lines in vitro (note: this cell line express both human and cynomolgus CD79b at similar levels). CD8+ T cells isolated from two healthy donors and BJAB cells (E:T ratio = 5:1) were incubated for 24 h with various concentrations of anti-cyCD79b/CD3 TDB and anti-CD79b/CD3 TDBs, as indicated.

We tested the clinical candidate and cynomolgus surrogate CD79b TDBs for in vitro cell killing, using transfected BJAB (BJAB.PD.cyCD79b.E3; 2 donors) cells. The BJAB cell lines expressed both huCD79b and cyCD79b at similar levels. The TDBs reduced the number of target cells in a dose-dependent manner, with potency (EC_50_; half-maximal effective concentration) in the low ng/mL range. EC_50_ values were found to range from 1.0 to 13 ng/mL, and from 0.31 to 2.4 ng/mL following anti-CD79b/CD3 and anti-cyCD79b.CD3 TDBs treatment, respectively (Figure 2).

### 3.3. Anti-CD79b/CD3, Cynomolgus Surrogate Anti-cyCD79b/CD3, and Anti-gD/CD3-High Affinity TDBs Exhibit Comparable PK in SCID.Beige Mice

**Figure 3:**
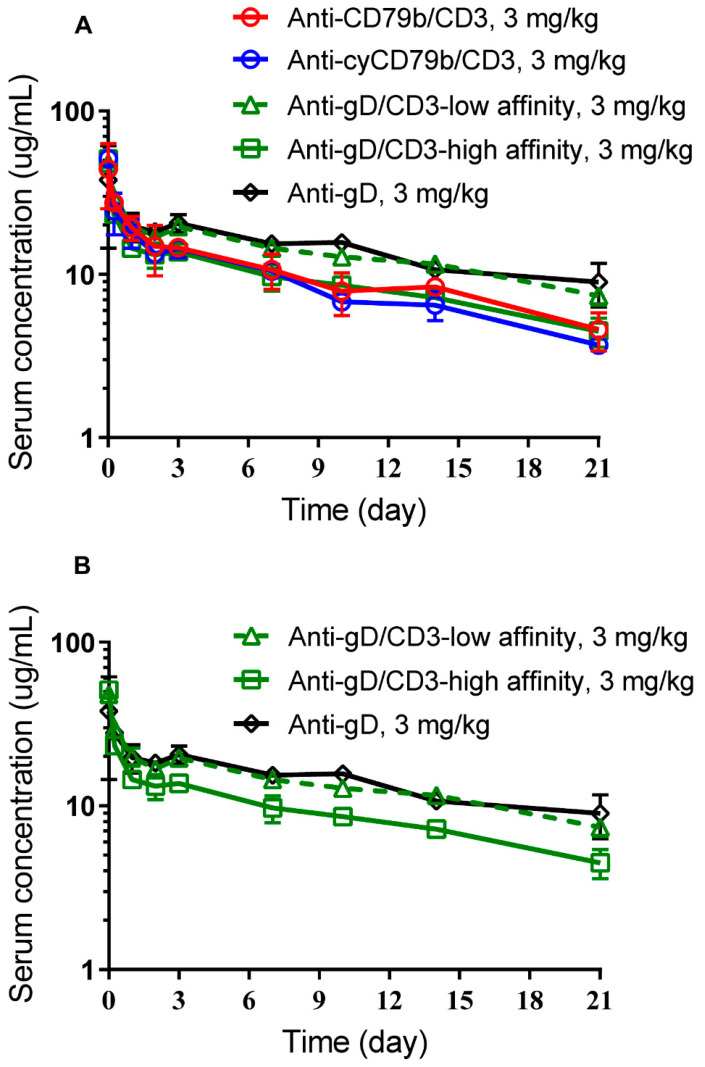
PK characterization of anti-CD79b/CD3 TDBs in SCID mice following a single IV bolus administration. (**A**) PK profiles in SCID.bg mice of clinical candidate anti-CD79b/CD3 (red circle, solid red line), cynomolgus surrogate anti-cyCD79b/CD3 (blue circle, solid blue line), or controls anti-gD (monospecific; black diamond, solid black line) and anti-gD/CD3-low affinity (bispecific; green triangle, dashed green line) and anti-gD/CD3-high affinity (bispecific; green square, solid green line). (**B**) Comparative PK profiles of bispecific anti-gD/CD3-low affinity and anti-gD/CD3-high affinity versus monospecific anti-gD in SCID.bg mice. Samples were collected using sparse sampling approach from n = 9 mice per group (n = 3 mice/timepoints) and data are presented here are group mean (±SD) for each treatment group.

Anti-CD79b/CD3, cynomolgus surrogate, anti-cyCD79b/CD3, and the monospecific (anti-gD) and bispecific controls (anti-gD/CD3-low affinity and -high affinity) are not cross-reactive antibodies in SCID.beige (SCID.bg) mice. Thus, target-independent clearances (CL) of these antibodies were characterized and compared in SCID.bg mice. The PK profiles of anti-gD/CD3-low affinity, anti-gD/CD3-high affinity, and the anti-gD control following a single IV bolus dose of 3 mg/kg are shown in Figure 3A,B, and the PK parameters are summarized in Table 2. Anti-gD/CD3 TDB with the high affinity CD3 arm showed slightly lower exposure compared to the anti-gD control antibody (Table 2), while anti-gD/CD3 TDB with the low affinity CD3 arm showed comparable PK to the anti-gD control. Systemic CL of anti-gD/CD3-low affinity was 6.9 mL/day/kg compared to higher CL of 10.9 mL/day/kg for anti-gD/CD3-high affinity, while the CL of the anti-gD control was 5.8 mL/day/kg. Anti-CD79b/CD3 TDB and its surrogate anti-cyCD79b/CD3 TDB (both TDBs containing the high affinity CD3 arm) showed lower exposure and faster systemic CL (range: 10–12 mL/day/kg) compared to the anti-gD control (5.8 mL/d/kg) (Table 2, Figure 3A). Since these antibodies are not cross-reactive with mouse and the antibodies with the high affinity CD3 arm, all had faster CL and lower exposure compared to the anti-gD control antibody; these results suggest that faster non-specific clearance of the anti-CD3-high affinity arm is likely due to the higher Fv charge on the high affinity CD3 arm, as previously described [40].

### 3.4. Impact of CD3 Binding Affinity on Anti-gD/CD3 TDB PK in Cynomolgus Monkeys

**Figure 4:**
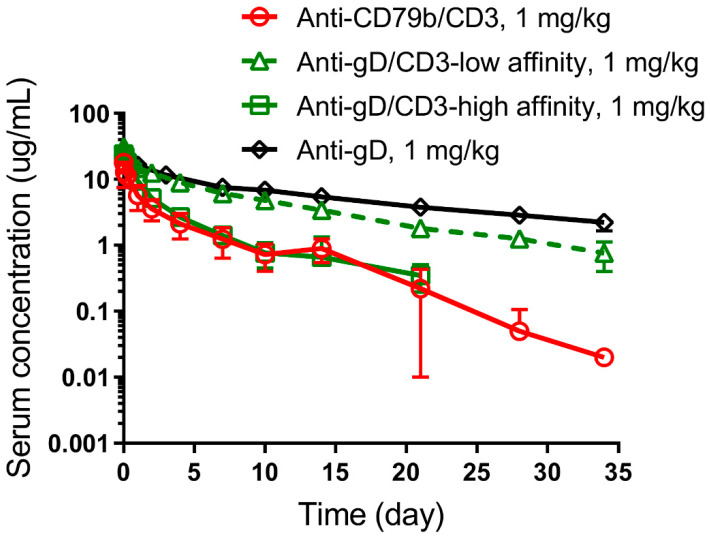
Impact of CD3 binding affinity on PK in cynomolgus monkey: Single dose PK profiles of anti-gD/CD3-low affinity (green triangle, dashed green line), anti-gD/CD3-high affinity (green square, solid green line), and clinical candidate anti-CD79b/CD3 (red circle, solid red line) in comparison to anti-gD control (black diamond, solid black line), following 3 mg/kg IV bolus administration in cynomolgus monkeys. Faster CL for both anti-CD79b/CD3 and anti-gD/CD3-high affinity compared to anti-gD/CD3-low affinity and anti-gD is attributed to higher-than-expected Fv charge in CD3 arm, in addition to high affinity CD3 binding. The lower limit of quantitation (LLOQ) was 1.56 ng/mL.

Bispecific anti-gD/CD3 control antibodies are cross-reactive with cynomolgus CD3ε. Anti-gD/CD3 TDBs with low (387 nM) or with high (12.8 nM) affinity CD3 arms were used to test the impact of CD3 binding on PK and PD. Cynomolgus monkeys were administered intravenously (IV) with a 1 mg/kg dose of either anti-gD/CD3-low affinity or anti-gD/CD3-high affinity, and serum PK was measured up to day 34 of post-treatment in these animals. Figure 4 shows the PK comparison between the two anti-gD/CD3 TDBs and historical anti-gD data at a comparable dose. NCA parameters of both anti-gD/CD3 antibodies, human anti-CD79b/CD3, and historical data for anti-gD antibody are shown in Table 3. In this regard, while anti-gD/CD3 with the high affinity CD3 arm showed a slightly lower maximum observed concentration (C_max_ = 24.0 ± 1.05 µg/mL) compared to C_max_ for the anti-gD/CD3-low affinity TDB (31.1 ± 4.70 µg/mL), both were within the C_max_ range expected for typical IgG1 molecules in cynomolgus, assuming a blood volume of 40–50 mL/kg [41]. Anti-gD/CD3 TDB with the high affinity CD3 arm showed faster CL and lower exposure compared to the anti-gD control antibody. Systemic CL of anti-gD/CD3-high affinity was 21.3 ± 4.2 mL/day/kg, compared to the considerably lower CL of anti-gD/CD3-low affinity (6.5 ± 0.94 mL/day/kg) and the anti-gD control (3.9 ± 0.62 mL/day/kg). Anti-gD/CD3 with the low affinity CD3 arm exhibited slightly higher CL than anti-gD, however, it was within a CL range expected for a typical IgG (<8 mL/day/kg) [42]. We also characterized the PK of human anti-CD79b/CD3 TDB in this single-dose cynomolgus study, following 1 mg/kg IV administration. Clinical candidate, anti-CD79b/CD3, showed slightly faster CL compared to anti-gD/CD3-high affinity (30.9 ± 13 vs. 21.3 ± 4.2 mL/day/kg). The inter-animal variability of anti-CD79b/CD3 PK profiles was high, likely due to the impact of anti-drug antibodies (ADA), as exemplified by the substantially reduced exposure in one animal with pre-existing ADA (discussed below). Observed mean C_max_ and AUC_0–7_ for anti-CD79b/CD3 were slightly lower compared to anti-gD/CD3-high affinity TDB. Faster CL for both anti-CD79b/CD3 and anti-gD/CD3-high affinity is likely attributable to highly positive Fv charge in the CD3 arm [40] in addition to high affinity CD3 binding. Since the anti-CD79b arm of human TDB candidate is not cross-reactive to cynomolgus CD79b, the target arm is not expected to have any impact upon CL. ADA was measured in serum samples for both anti-gD/CD3 TDBs and anti-CD79b/CD3 TDB from day 14 to day 34 post-dose (Table S1).

All animals (100%) that received anti-gD/CD3-high affinity and anti-CD79b/CD3 TDBs exhibited high titers of ADA by day 14. Pre-dose samples from one anti-CD79b/CD3-treated animal (1003) were ADA positive and showed reduced exposure compared to the other two animals (Table S1). The anti-gD/CD3-low affinity TDB was less immunogenic, with no observed ADA at day 14. Only one out of three animals developed ADA by day 21 post-dose, and even this animal showed only low-titer ADA, and no significant impact on serum PK was observed.

### 3.5. Surrogate (Anti-cyCD79b/CD3) TDB Exhibited Target-Dependent Clearance in Cynomolgus Monkey

**Figure 5:**
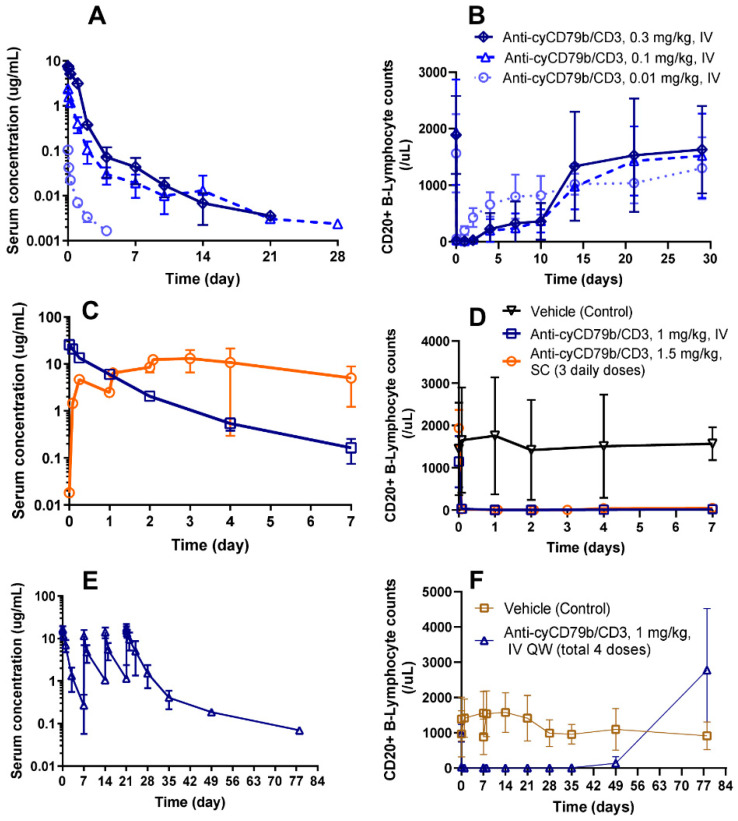
Surrogate TDB (anti-cyCD79b/CD3) was well tolerated and showed potent B cell depletion, exhibited rapid and target mediated clearance; (**A**) PK profiles of anti-cyCD79b/CD TDB up to 28 days, following a single IV bolus dose in cynomolgus monkeys at doses of 0.01, 0.1, and 0.3 mg/kg (n = 4). (**B**) Dose-dependent CD20+ B lymphocyte changes (absolute numbers) in cynomolgus blood following single IV bolus of the anti-cyCD79b/CD3 TDB at 0.01, 0.1, and 0.3 mg/kg (n = 4). (**C**) PK profiles of anti-cyCD79b/CD3 TDB in cynomolgus monkeys, following single 1 mg/kg IV bolus injection or three daily 1.5 mg/kg SC doses (n = 3). (**D**) Robust B cell (CD20+ B lymphocyte) depletion (absolute numbers) in cynomolgus blood following single 1 mg/kg IV bolus injection or three daily 1.5 mg/kg SC doses of anti-cyCD79b/CD3 TDB (n = 3). (**E**) PK profiles of anti-cyCD79b/CD3 TDB following weekly IV administration at 1mg/kg (total 4 doses) in a four-week repeat-dose cynomolgus GLP study with a seven-week recovery period (n = 10; five males, five females). (**F**) Pronounced B cell (CD20+ B lymphocyte) depletion (absolute numbers) in cynomolgus blood following weekly IV administration of anti-cyCD79b/CD3 TDB at 1 mg/kg (total 4 doses) in a four-week repeat-dose cynomolgus GLP study with a seven-week recovery period (n = 10; five Males, five Females).

In order to assess the PKPD of targeting CD79b with a T cell-recruiting bispecific antibody in non-human primates, a surrogate anti-cyCD79b/CD3 TDB with comparable in vitro potency was produced with a target arm that recognizes cynomolgus monkey CD79b and it has the same high affinity as in humans for the anti-CD3 arm. Figure 5A shows PK profiles of anti-cyCD79b/CD3 in cynomolgus monkeys, following a single IV bolus dose of 0.01, 0.1, or 0.3 mg/kg. PK parameters after NCA are summarized in Table 4. The PK profiles showed a greater than dose proportional increase in C_max_ and AUC_0–7_ between 0.01 mg/kg to 0.3 mg/kg, whereas the increase in C_max_ was approximately dose proportional between 0.1 to 0.3 mg/kg. Mean total CL in groups dosed at 0.01, 0.1, and 0.3 mg/kg were 315 ± 80.9, 66.2 ± 16.6, and 41.8 ± 5.1 mL/day/kg, respectively. The volume of distribution at steady-state (V_ss_) ranged from 43.3 to 284 mL/kg at the doses tested. Both total CL (~7.6 fold) and V_ss_ (~6.6-fold) decreased with increasing dose, suggesting target-mediated drug disposition. These data indicate that anti-cyCD79b/CD3 TDB exhibited nonlinear pharmacokinetics consistent with the expected target-mediated CL, due to binding to both cynomolgus CD79b and CD3 on the target cells.

In addition, we measured B cells in blood to evaluate the PKPD relationship of anti-cyCD79b/CD3 TDB in cynomolgus monkeys administered a single dose of 0.01, 0.1, and 0.3 mg/kg. The temporal profile of B cell counts is shown in Figure 5B. We observed a dose-dependent decrease in B cells, and B cell counts in peripheral blood dropped rapidly after anti-cyCD79b/CD3 TDB administration (2 h post-dose) for each treatment group. The B cell counts exhibited a trend to recovery after 24 h for the 0.01 mg/kg group, and after day 8 for the 0.1 mg/kg and 0.3 mg/kg groups. Generally, all the groups reached a complete recovery by day 29 post-dose.

### 3.6. Single- and Multiple-Dose PKPD of Anti-cyCD79b/CD3 TDB in Cynomolgus Monkeys

In a one-week single dose study, we characterized the PKPD of surrogate anti-cyCD79b/CD3 TDB in cynomolgus monkeys at 1 mg/kg IV bolus injection or after three daily 1.5 mg/kg SC doses (Figure 5C,D). The SC administration route was being investigated as a potential alternative to IV dosing to reduce patient burden; therefore, we sought to understand the PKPD profile as well as safety relative to IV administration. The observed C_max_ values were approximately 40% lower with three daily SC doses of 1.5 mg/kg compared to a single IV dose of 1 mg/kg (mean C_max_ = 15.4 ± 6.61 and 25.8 ± 2.84 µg/mL, respectively). We observed greater exposure, as defined by area under the serum concentration-time curve from the end of infusion to the last measurable time point (AUC_0–last_), after three daily SC doses than after a single IV dose (Table 4; 58.6 ± 34.5 and 19.7 ± 0.95 day·µg/mL, respectively). Following SC administration, the mean time of the observed C_max_ (t_max_) was 3.03 ± 0.96 days. B cell counts in circulation were profoundly decreased after dosing for both dose groups. No B cell recovery was observed for up to seven days (until the end of the study), following both IV and SC dose groups (Figure 5D). As expected, B cell counts were unaltered in the control treated group.

### 3.7. PKPD of Surrogate (Anti-cyCD79b/CD3) TDB in a Four-Week Repeat Dose GLP Study (with Seven-Week Recovery) in Cynomolgus Monkeys

The serum concentration of anti-cyCD79b/CD3 in female and male cynomolgus monkeys, following weekly IV administration of 1 mg/kg drug, was measured in a four-week repeat-dose cynomolgus study with a seven-week recovery period (Figure 5E). The exposure of anti-cyCD79b/CD3 was generally higher after the last dose compared to the first dose, except for one animal (2501), where the exposure of anti-cyCD79b/CD3 decreased (Table 5). This animal (2501) developed high titer (4.23) ADA on day 28 (Table S2). No sex differences in exposure (AUC_0–7_) were observed after first dose. Conversely, a sex difference was observed after the fourth dose (AUC_21–28_), and exposure in males was higher than in females (Table 5). Anti-cyCD79b/CD3 was generally not quantifiable at TK day 49 (recovery phase) except in one animal, which showed a detectable concentration up to day 77. Nine out of ten treated animals tested positive for ADA (incidence: 90%), on one or more occasions post-dose. There was not an obvious correlation with the reduced systemic exposure of anti-cyCD79b/CD3 on TK day 14 and day 28 and during the recovery period (Table S2). Four out of ten animals in the control group had one or more confirmed positive ADA samples post-dose, and no treated animals were confirmed positive for ADA prior to dosing. ADA positivity in the control group cannot be explained but has been observed previously [43], and it does not preclude an assessment of the impact of ADA positivity on anti-cyCD79b/CD3 exposure in the treated groups post-dose. A robust reduction in B cell counts relative to both pre-dose and time-matched control values was noted for all anti-cyCD79b/CD3-dosed animals, with a nadir by day 1 that was sustained through day 49 (Figure 5F). The B lymphocyte absolute counts were increased on day 78, compared to the baseline (pre-dose) average values. As expected, no impact on B cell counts was observed in the vehicle control group.

### 3.8. Bispecific TMDD Model Characterized Anti-cyCD79b/CD3 Disposition Kinetics and Peripheral Blood B Cell Decrease

The bispecific TMDD model development for anti-cyCD79b/CD3 TDB was based on three studies in cynomolgus monkeys (28 representative monkeys PKPD profiles) spanning a dose range of 0.01–1 mg/kg (single IV dose), 1.5 mg/kg daily (three SC doses), and 1 mg/kg weekly (four total IV doses followed by a seven-week recovery). Figure 1 shows the schematic of the bispecific TMDD model, which includes PK of anti-cyCD79b/CD3 TDB and changes in B cell counts after anti-cyCD79b/CD3 administration, as the drug effect (details of its development process is presented in the Section 2).

**Figure 6:**
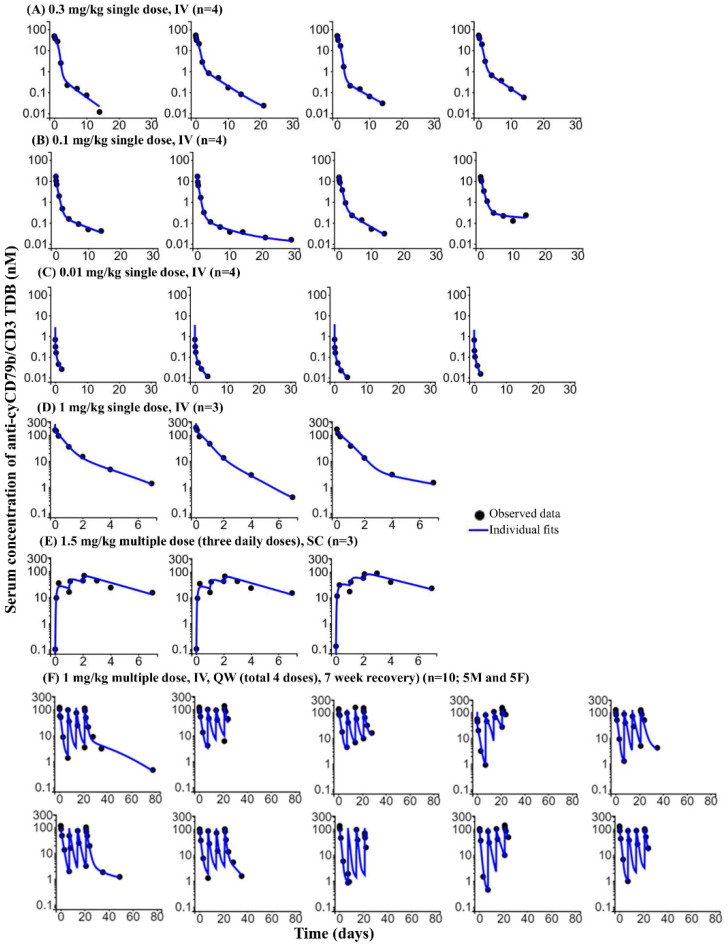
Bispecific TMDD model-based fitting of monkey PK data for surrogate (anti-cyCD79b/CD3) TDB. Individual PK profiles for 28 representative cynomolgus monkeys receiving anti-cyCD79b/CD3 TDB; 0.3 mg/kg IV PK profiles (**A**), 0.1 mg/kg IV PK profiles (**B**), 0.01 mg/kg IV PK profiles (**C**), 1 mg/kg IV PK profiles (**D**), 1.5 mg/kg, SC PK profiles (**E**) and 1 mg/kg IV, QW (total 4 doses with 7-week recovery) PK profiles (**F**) [top row represents male PK profiles and bottom row represents females PK profiles]. Circles represent the observed data. Solid blue lines are the individual model predictions.

Figure 6 shows the time courses of observed and predicted anti-cyCD79b/CD3 TDB concentrations. The PK profiles of anti-cyCD79b/CD3 TDB were best described by a two-compartment model with linear and target-mediated elimination pathways. Estimated population pharmacokinetic parameters and residual errors are reported in Table 6. The majority of the parameter estimates were obtained with reasonable precision (%RSE < 50%) across three different studies. The only exception was the estimates of k_int_complex3_, with a %RSE of >50%. The estimated mean non-specific CL (20 mL/day/kg) was in line with the observed clearance and consistent with slightly higher range of non-specific CL reported for bispecific antibodies [5,44]. The central volume of distribution (51 mL/kg) was close to the reported physiological blood volume of cynomolgus monkeys [41]. Distribution CL and absorption rate constant (k_a_; SC administration) were comparable to the observed values for other bispecific and human IgG1 antibodies in monkeys [5,44,45] (Table 6). The model-fitted curves captured the data well and yielded unbiased and precise curve fits (Figure 6, Figures S1 and S2).

**Figure 7:**
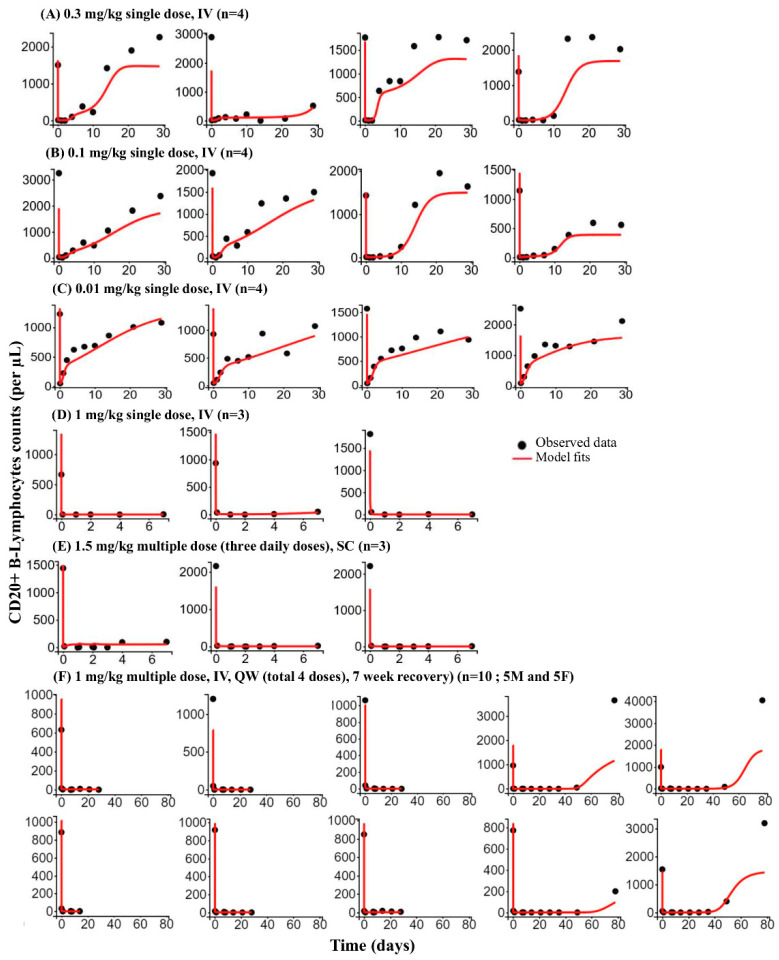
Bispecific TMDD model-based fitting of monkey PD data for surrogate (anti-cyCD79b/CD3) TDB. Individual B cell (CD20+ B lymphocyte) counts changes profiles for 28 representative cynomolgus monkeys receiving anti-cyCD79b/CD3 TDB. 0.3 mg/kg IV PD profiles (**A**), 0.1 mg/kg IV PD profiles (**B**), 0.01 mg/kg IV PD profiles (**C**), 1 mg/kg IV PD profiles (**D**), 1.5 mg/kg, SC PD profiles (**E**), and 1 mg/kg IV, QW (total 4 doses with 7-week recovery) PD profiles (**F**) [top row represents male PD profiles and bottom row represents females PD profile]. Black circles represent the observed data. Solid red lines are the individual model predictions.

B cell count changes were described, with an indirect response model integrated with the developed bispecific TMDD model for anti-cyCD79b/CD3 TDB (Figure 1). The time course of observed and predicted B cell counts (individual fits) is shown in Figure 7. The proposed PD model can predict the general trend of dose-dependent B cell depletion and recovery for each dose group; however, the model could not capture the B cell counts on day 14 onwards in the two animals from the 0.3 mg/kg dose group, likely due to the rebound effect (i.e., B cell counts increased above pre-dose levels). The elimination half-life of B cells was fixed to 55 days, based on the literature-reported value [38] (Table 6). The estimated population mean of the baseline count was consistent with observed pre-dose B cell counts in cynomolgus blood. All estimated parameters are reported in Table 6. The model-fitted curves captured all of the B cell count profiles well, and all model parameters were estimated with reliable precision (Figure 7, Figures S1 and S3). The coefficient of correlation (r) for the observed versus population fitted profiles was ≥0.959 and ≥0.886 for serum concentrations and B-cell counts profiles, respectively (Figure S1).

## 4. Discussion

Human biotherapeutic agents display a high degree of species specificity, and identifying a pharmacologically relevant non-clinical species for PKPD and safety assessment that translates to humans remains a challenge. Nevertheless, several successfully marketed biotherapeutic agents used mouse or cynomolgus surrogate molecules to evaluate non-clinical PKPD and safety in longer-term general toxicity and developmental and reproductive toxicity studies, including polatuzumab, efalizumab, infliximab, and IFN-γ [39,46,47,48,49]. Surrogate molecules are typically considered when the clinical candidate is pharmacologically active only in humans and when the presence of anti-drug antibodies can affect drug exposure, thereby limiting the ability to conduct a thorough PKPD and safety evaluation [49,50].

In the case of the anti-CD79b/CD3 bispecific antibody explored in this study, a three amino acid difference in the human and cynomolgus monkey CD79b receptor conferred selective binding of the clinical antibody to human CD79b only [30,39]. Therefore, we generated an anti-cyCD79b/CD3 surrogate TDB that binds to a similar epitope on CD79b in cynomolgus monkeys as a surrogate clinical antibody, with which to evaluate anti-CD79b-mediated in vivo pharmacology prior to entry into humans [18].

We hypothesized that CD3 affinity would be a key driver of both efficacy and safety. Thus, we evaluated anti-gD/CD3 TDBs with low or with high CD3 affinity and a monospecific control in SCID.bg mice (a non-binding species), before evaluating the PKPD behavior of these antibodies in cynomolgus monkeys (binding species). We selected an immune-deficient mouse strain, SCID.bg, to avoid any potential ADA impact on the exposure of the TDBs. TDBs containing a high affinity CD3 arm appeared to clear more rapidly than the anti-gD antibody, even in the non-binding species (mouse). This observation is consistent with the previously reported clearance of an anti-CLL/CD3 TDB, with varying CD3 arm affinities in SCID.bg mice [24,40]. A positively charged patch in the Fv region of the anti-CD3-high antibody is potentially associated with the higher CL of this TDB [40,51], and might contribute to the increased CL of both anti-CD79b/CD3 and anti-gD/CD3, compared to the bivalent anti-gD lacking a CD3 arm. In cynomolgus monkeys, the PK of these TDBs behaved in a CD3-affinity-dependent manner. The anti-gD/CD3 TDB with a high-affinity CD3 arm (K_d_ = 12.8 nM) exhibited faster clearance and lower drug exposure compared with the low CD3 affinity anti-gD/CD3 TDB (K_d_ = 387 nM). These results are consistent with the PK behavior of CLL-1/TDB containing low or high affinity CD3 arms in cynomolgus monkeys [24]. Anti-gD/CD3 TDB with a high affinity CD3 arm also showed faster CL and lower exposure, compared to anti-gD control antibody at 1 mg/kg. This faster CL potentially reflects both the impact of the positively charged patch on the CD3 arm as well as the increased binding to the CD3 arm due to higher affinity [40]. In this regard, a dose-ranging study is needed to determine non-specific CL of the anti-gD/CD3 TDB containing the high affinity CD3 arm. Taken together, these results demonstrate that an understanding of the PKPD behavior of CD3-containing monovalent control molecules is critical in designing cynomolgus safety studies to inform the development of TDBs. Future work with engineered monovalent control molecules with variable CD79b affinities may provide an additional insight into their in vivo pharmacology, study design for multi-dose cynomolgus safety studies, and further development of bispecific antibodies.

The current study also evaluated anti-CD79b mediated in vivo pharmacology in cynomolgus monkeys using an anti-cyCD79b/CD3 surrogate bispecific antibody (containing a high affinity CD3 arm) that binds to a similar epitope as the clinical antibody. In cynomolgus monkeys, the anti-cyCD79b/CD3 TDB exhibited nonlinear pharmacokinetics consistent with target-mediated CL, likely due to target CD79b antigen internalization and enhanced binding to CD3. The observed rapid and dose-dependent CL and shorter elimination of anti-cyCD79b/CD3 were comparable with that of other TDBs [24,25,40,52] in cynomolgus monkeys, as expected based upon drug structure. In addition, anti-cyCD79b/CD3 TDB resulted in an anticipated dose-dependent decrease in B cells that was consistent with the molecule’s mechanism of action [18]. B cell depletion was achieved in all animals at low doses, suggesting a potent anti-target activity of anti-cyCD79b/CD3. Notably, the duration of this decrease in B cell numbers was variable within the dose groups, and the recovery of B cells was rapid at lower anti-cyCD79b/CD3 doses. At the lowest dose tested (0.01 mg/kg), B cell counts were minimal immediately after anti-cyCD79b/CD3 TDB administration, but began to increase after day 1, likely reflecting a low cell-killing activity at lower concentrations. This dose-ranging PKPD study, including low doses, enabled us to define the PKPD relationship.

We incorporated an anti-cyCD79b/CD3 binding to both CD3 and CD79b in the PK model as a target-mediated disposition process. We assumed that anti-cyCD79b/CD3 binds to CD3 and CD79b to form binary complexes, followed by further cross-linking with either CD3 or CD79b to form a ternary complex, as reported for other bispecific antibodies [4]. For many large molecule therapeutics, including bispecific antibodies, binding to the pharmacologic target influences drug distribution and elimination, and can result in nonlinear pharmacokinetics [5,44,45,53]. Model estimates (k_int___complex2_ and k_int_complex3_, Table 6) indicated that a relatively faster internalization rate of CD79b complex and ternary complex could likely contribute to target-mediated CL of anti-cyCD79b/CD3, in addition to enhanced CD3 binding. Schropp et al. [7], demonstrated that faster internalization of target receptor and ternary complex lead to faster elimination of bispecific antibodies and to nonlinear PK. Due to the cross-linking nature of binding kinetics (i.e., both binary complexes further cross-link with the other target forming the ternary complex; see Figure 1), the functional ternary complex (i.e., assumed to be drivers of changes in B cell counts in the model) shows different PK properties, as compared to the classical concentration effect relationship [7]. The model predicts a delayed buildup of ternary complex concentrations for increasing doses. The predicted ternary complex concentrations are substantially lower compared to very high serum concentrations at respective doses, however, they are sufficient to drive B cell depletion (Figure S4). This is consistent with an in vitro study conducted by co-culturing Jurkat T cells and Daudi B cells in the presence of CD19/CD3 bispecific antibody, where a decrease in ternary complex concentration was observed at higher concentrations of the drug [54]. The turnover of B cells was characterized by zero-order cell proliferation and first-order decay over time, as a simplified representation of the underlying sequential development steps of B cell maturation in bone marrow [55]. The dose-dependent depletion and recovery of B cells was captured by the model, with the exception of a few points (days 14 and 21) in 0.3 mg/kg dose group. In the four-week repeat dose study, B cell depletion and recovery was well described by the model; however, the model could not capture B cell counts at day 78 in a few animals. In these animals, B cell counts reached above pre-dose levels, consistent with rebound phenomenon. The rebound of B cells may be attributed to hematopoiesis that occurred on day 14 in a few animals from the 0.3 mg/kg dose group and on day 78 in few animals from the 1 mg/kg repeat dose group. Extending the study and measuring B cell at later time points may have shown B cells returning to baseline. Additionally, no B cell counts were available post-day 7 in the 1 mg/kg IV and 1.5 mg/kg SC dose groups. Thus, the target cell rebound phenomenon was not incorporated into the model. Further evaluation of anti-CD79b/CD3 in an extended duration PKPD study is warranted, to elucidate the dynamics of target cell rebound using a mechanistic PKPD model.

Minimal, physiologically based PKPD models and pharmacology models for quantitative systems, which include mechanistic and systemic information, have been used to describe PKPD as well as relevant safety and efficacy markers such as T cell activation, cytokine increases, and cell killing in vivo [6,16,36]. These models often include the binding kinetics of ternary complex formation (i.e., of the drug and its two target cells form a trimer or a synapse that trigger cell killing), coupled with the drug’s in vitro potency in these models [6,16,36]. T cell activation, target dynamics, and cell trafficking information are used to calibrate such models, depending on the system used (i.e., mice, non-human primates, or humans). These models help elucidate mechanisms behind safety and efficacy and capture T cell activation, cytokine release, and target cell killing, to enable concurrent prediction of efficacy- and safety-related biomarkers.

On the other hand, our integrated semi-mechanistic PKPD model, while limited in scope, offers opportunities to aid in bispecific antibody drug discovery, for example, in early development stages when there is limited data (e.g., design and dose selection in multiple dose safety pharmacology and toxicity studies) and at the beginning of clinical development. A limitation of the model is that complex T cell dynamics, trafficking, and activation driving the peripheral depletion of B cells were not incorporated into the model. However, we incorporated the sequential binding of surrogate anti-cyCD79b/CD3 to both CD3 and CD79b as an essential step to describe the mechanism of action of the bispecific antibody, as the starting point of its pharmacological activity is the formation of the ternary complex between anti-cyCD79b/CD3, CD3, and CD79b. Our model with these features (e.g., sequential binding and ternary complex formation) was able to adequately characterize the PK and B cell changes in cynomolgus monkeys, following IV and SC administrations, and can be leveraged to design and select dose for future preclinical and clinical studies.

## 5. Conclusions

In summary, a surrogate anti-cyCD79b/CD3 TDB was highly effective in killing CD79b-expressing B cells in vitro, well tolerated in cynomolgus monkeys, and exhibited dose-dependent B cell depletion and recovery as well as nonlinear pharmacokinetics consistent with target-mediated clearance. Our model yielded unbiased and precise curve fits for PKPD data, following IV and SC administration of anti-cyCD79b/CD3 TDB. Modeling results indicated that anti-cyCD79b/CD3 TDB’s rapid and target-mediated clearance may be attributed to faster internalization of CD79b, in addition to enhanced CD3 binding. Overall, this study highlights the utility of using a surrogate drug candidate to assess the PKPD and pharmacology of a novel molecule, and emphasizes the complex interaction between TDBs and their targets. Taken together, the information gained from this study may be applicable to the development of other biotherapeutics.

## Figures and Tables

**Table 1:** Binding affinity (Biacore measurement made at 37 °C) for the component antibodies used to make bispecific in this study.

Monovalent Affinity	CD79b ^#^, K_D_ (nM)		CD3ε [25], K_D_ (nM)	
	Human	Cynomolgus	Human	Cynomolgus
Anti-CD79b	17 *	-	14.4	12.8
Anti-cyCD79b	-	1 *	14.4	12.8
Anti-CD3-low affinity	-	-	446	387
Anti-CD3-high affinity	-	-	14.4	12.8

^#^ CD79b epitope peptide sequence ([30,39]): human CD79b: A**R**SED**R**Y**R**NPKGS; cyno CD79b: A**K**SED**L**Y**P**NPKGS. Sequence differences are indicated in bold and underlined. * measurements made at 25 °C.

**Table 2:** Summary of mean PK parameters following a single IV administration of TDBs in SCID.bg mice.

	SCID.bg Mice				
PK Parameter	Anti-CD79b/CD3	Anti-cyCD79b/CD3	Anti-gD/CD3-Low Affinity	Anti-gD/CD3-High Affinity	Anti-gD
	(3 mg/kg)	(3 mg/kg)	(3 mg/kg)	(3 mg/kg)	(3 mg/kg)
C_max_^a^ (μg/mL)	44.2 ± 10.9	51.2 ± 2.2	47.9 ± 1.1	50.9 ± 2.0	37.9 ± 13.5
AUC_0–last_ (μg·day/mL)	215.1	193.4	289.2	197.6	304.9
AUC_0–∞_ (μg·day/mL)	292.5	246.0	432.5	274.8	518.4
CL (mL/kg/day)	10.3	12.2	6.93	10.9	5.79
V_ss_ (mL/kg)	161	162.4	132.2	180	136.1

^a^ Reported parameter variability in C_max_ represents standard error of mean (SEM) and is result of naïve pool approach with NCA. The plasma concentration vs. time data was naïve pooled together (sparse sampling approach) to provide on PK parameter estimate for each treatment group. Therefore, SD is not provided, and the reported mean parameter represents the composite value from the naïve pooling of animals.

**Table 3:** Summary of mean (±SD) PK parameter following a single IV (1 mg/kg) administration of TDBs in cynomolgus monkeys.

PK Parameter	Anti-CD79b/CD3	Anti-gD/CD3-Low Affinity	Anti-gD/CD3-High Affinity	Anti-gD
C_max_ (μg/mL)	17.8 ± 5.3	31.1 ± 4.7	24.0 ± 1.05	27.6 ± 3.9
AUC_0–7_ (μg·day/mL)	25.1 ± 8.6	78.2 ± 4.9	36.9 ± 2.1	86.7 ± 2.2
AUC_0–last_ (μg·day/mL)	36.2 ± 14.0	146 ± 18.1	46.1 ± 7.5	206 ± 17.8
AUC_0–∞_ (μg·day/mL)	36.3 ± 14.1	157 ± 24.9	48.1 ± 8.6	260 ± 42.4
CL (mL/kg/day)	30.9 ± 13.0	6.46 ± 0.94	21.3 ± 4.2	3.9 ± 0.62
V_ss_ (mL/kg)	154 ± 34.0	72.5 ± 8.1	94.5 ± 32.8	80.1 ± 4.7

**Table 4:** Summary of mean (±SD) PK parameter of surrogate anti-cyCD79b/CD3 TDB following a single- or repeat-dose administration to cynomolgus monkeys.

PK Parameter	Anti-cyCD79b/CD3				
	Dose Ranging PK (Single Dose)			Single (IV) or Multiple (SC) Dose 1-Week TK	
	(0.01 mg/kg)	(0.1 mg/kg)	(0.3 mg/kg)	(1 mg/kg)	(1.5 mg/kg, daily, SC × 3)
C_max_ (μg/mL)	0.10 ± 0.0029	2.4 ± 0.11	7.6 ±0.30	25.8 ± 2.8	15.4 ± 6.6
AUC_0–7_ (μg·day/mL)	0.030 ± 0.007	1.5 ± 0.38	7.1 ± 0.80	19.7 ± 0.95	58.6 ± 34.5
AUC_0–last_ (μg·day/mL)	0.030 ± 0.0068	1.5 ± 0.38	7.2 ± 0.82	19.7 ± 0.95	58.6 ± 34.5
AUC_0–∞_ (μg·day/mL)	0.033 ± 0.0074	1.6 ± 0.41	7.3 ± 0.81	NA	NA
CL (mL/kg/day)	315 ± 80.9	66.2 ± 16.6	41.8 ± 5.1	50.0 ± 2.3	NA
V_ss_ (mL/kg)	284 ± 52.4	165 ± 105	43.3 ± 10.1	57.2 ± 9.3	NA
T_max_ (day)	NA	NA	NA	NA	3.0 ± 0.96

NA: Not applicable.

**Table 5:** Summary of PK parameters of surrogate anti-cyCD79b/CD3 TDB, following weekly IV administration of 1 mg/kg in a four-week repeat-dose cynomolgus study with a seven-week recovery period (data are represented as mean ± SD).

	C_max Day 0_ (µg/mL)	C_max Day 21_ (µg/mL)	AUC_0–7_ (μg·day/mL)	AUC_21–28_ (μg·day/mL)	AUC_0–last_ (μg·day/mL)	Accumulation Ratio
Male	16.9 ± 4.9	20.5 ± 2.8	23.7 ± 8.96	55.5 ± 22.5	148 ± 30.2	2.34
Female	17.6 ± 2.4	15.5 ± 3.8	21.4 ± 3.5	29.3 ± 16.9	107 ± 23.1	1.37
Combined	17.2 ± 3.6	18.1 ± 4.1	22.5 ±6.5	42.4 ± 123.3	128 ± 33.5	1.88

**Table 6:** Pharmacokinetic/pharmacodynamic model parameter estimates for surrogate anti-cyCD79b/CD3 TDB in cynomolgus monkeys.

Parameter	Definition	Unit	Estimate (%RSE)	Source
CL	Clearance	mL/day/kg	20.0 (47.3)	Model estimated
CLd	Distribution clearance	mL/day/kg	22.0 (16.0)	Model estimated
V1	Central volume of distribution	mL/kg	51.0 (6.59)	Model estimated
V2	Peripheral volume of distribution	mL/kg	90.0 (fixed) [5]	
k_on_CD3_	CD3 association constant	1/nM/day	4.45	In vitro data
Kd__CD3_	CD3 binding affinity (k_off_/k_on_)	nM	12.8	In vitro data (Table 1)
k_on_CD79b_	CD79b associate constant	1/nM/day	2.96	In vitro data
Kd__CD79b_	CD79b binding affinity (k_off_/k_on_)	nM	1.0	In vitro data (Table 1)
ln(2)/k_deg_CD3_	Degradation half-life of CD3	day	0.74 (32.8)	Model estimated
ln(2)/k_deg_CD79b_	Degradation half-life of CD79b	day	0.79 (31.7)	Model estimated
ln(2)/k_int_complex1_	elimination half-life for complex1	day	346.5 (34.2)	Model estimated
ln(2)/k_int_complex2_	elimination half-life for complex2	day	7.79 (63.6)	Model estimated
ln(2)/k_int_complex3_	elimination half-life for complex3	day	5.77 (12.9)	Model estimated
k_a_	SC absorption rate constant	1/day	0.31 (14.7)	Model estimated
F	SC bioavailability	Fraction	0.84 (fixed)	[45]
E_max_	Maximum stimulation factor	unitless	221 (49.3)	Model estimated
EC_50_	Ternary complex concentration inducing 50% of k_out_	nM	1.20 (21.0)	Model estimated
BSL__B-lymphocyte_	Baseline of CD20+ B-lymphocyte	counts/µL	1223.3 (14.0)	Model estimated
ln(2)/k_out_B-lymphocyte_	Degradation half-life of B-lymphocyte	day	55 (fixed)	[38]

## Data Availability

The data presented in this study are available in the research article and supplementary material here.

## Supplementary Materials

The following supporting information can be downloaded at: https://www.mdpi.com/article/10.3390/pharmaceutics14050970/s1, Figure S1: Observed versus predicted serum concentration and B cell (CD20+ B lymphocyte) counts, following anti-CD79b/CD3 treatment in cynomolgus monkeys. Figure S2: Population PK fits in cynomolgus monkeys receiving anti-cyCD79b/CD3 TDB. Figure S3: Population PD (B cell counts) fits in cynomolgus monkeys receiving anti-cyCD79b/CD3 TDB. Figure S4: Model-predicted anti-cyCD79b/CD3 TDB serum concentration, ternary complex concentration, and B cell depletion. Table S1. Anti-drug antibody (ADA) titers against anti-CD79b/ CD3, anti-gD/CD3-low affinity, and anti-gD/CD3-high affinity TDBs. Table S2. Anti-drug antibody (ADA) titers against anti-cyCD79b/ CD3 TDB.

## References

[1] Baeuerle P.A., Reinhardt C. (2009). Bispecific t-cell engaging antibodies for cancer therapy. Cancer Res..

[2] Clevers H., Alarcon B., Wileman T., Terhorst C. (1988). The t cell receptor/cd3 complex: A dynamic protein ensemble. Annu. Rev. Immunol..

[3] Lejeune M., Köse M.C., Duray E., Einsele H., Beguin Y., Caers J. (2020). Bispecific, t-cell-recruiting antibodies in b-cell malignancies. Front. Immunol..

[4] Chudasama V.L., Zutshi A., Singh P., Abraham A.K., Mager D.E., Harrold J.M. (2015). Simulations of site-specific target-mediated pharmacokinetic models for guiding the development of bispecific antibodies. J. Pharmacokinet. Pharmacodyn..

[5] Campagne O., Delmas A., Fouliard S., Chenel M., Chichili G.R., Li H., Alderson R., Scherrmann J.M., Mager D.E. (2018). Integrated pharmacokinetic/pharmacodynamic model of a bispecific cd3xcd123 dart molecule in nonhuman primates: Evaluation of activity and impact of immunogenicity. Clin. Cancer Res. Off. J. Am. Assoc. Cancer Res..

[6] Betts A., Haddish-Berhane N., Shah D.K., van der Graaf P.H., Barletta F., King L., Clark T., Kamperschroer C., Root A., Hooper A. (2019). A translational quantitative systems pharmacology model for cd3 bispecific molecules: Application to quantify t cell-mediated tumor cell killing by p-cadherin lp dart^®^. AAPS J..

[7] Schropp J., Khot A., Shah D.K., Koch G. (2019). Target-mediated drug disposition model for bispecific antibodies: Properties, approximation, and optimal dosing strategy. CPT Pharmacomet. Syst. Pharmacol..

[8] Riethmüller G. (2012). Symmetry breaking: Bispecific antibodies, the beginnings, and 50 years on. Cancer Immun..

[9] Trivedi A., Stienen S., Zhu M., Li H., Yuraszeck T., Gibbs J., Heath T., Loberg R., Kasichayanula S. (2017). Clinical pharmacology and translational aspects of bispecific antibodies. Clin. Transl. Sci..

[10] Krishnamurthy A., Jimeno A. (2018). Bispecific antibodies for cancer therapy: A review. Pharmacol. Ther..

[11] Labrijn A.F., Janmaat M.L., Reichert J.M., Parren P. (2019). Bispecific antibodies: A mechanistic review of the pipeline. Nat. Rev. Drug Discov..

[12] Wu J., Fu J., Zhang M., Liu D. (2015). Blinatumomab: A bispecific t cell engager (bite) antibody against cd19/cd3 for refractory acute lymphoid leukemia. J. Hematol. Oncol..

[13] Goebeler M.E., Knop S., Viardot A., Kufer P., Topp M.S., Einsele H., Noppeney R., Hess G., Kallert S., Mackensen A. (2016). Bispecific t-cell engager (bite) antibody construct blinatumomab for the treatment of patients with relapsed/refractory non-hodgkin lymphoma: Final results from a phase i study. J. Clin. Oncol. Off. J. Am. Soc. Clin. Oncol..

[14] Gökbuget N., Dombret H., Bonifacio M., Reichle A., Graux C., Faul C., Diedrich H., Topp M.S., Brüggemann M., Horst H.A. (2018). Blinatumomab for minimal residual disease in adults with b-cell precursor acute lymphoblastic leukemia. Blood.

[15] Sun L.L., Ellerman D., Mathieu M., Hristopoulos M., Chen X., Li Y., Yan X., Clark R., Reyes A., Stefanich E. (2015). Anti-cd20/cd3 t cell-dependent bispecific antibody for the treatment of b cell malignancies. Sci. Transl. Med..

[16] Hosseini I., Gadkar K., Stefanich E., Li C.C., Sun L.L., Chu Y.W., Ramanujan S. (2020). Mitigating the risk of cytokine release syndrome in a phase i trial of cd20/cd3 bispecific antibody mosunetuzumab in nhl: Impact of translational system modeling. NPJ Syst. Biol. Appl..

[17] Pfeifer M., Zheng B., Erdmann T., Koeppen H., McCord R., Grau M., Staiger A., Chai A., Sandmann T., Madle H. (2015). Anti-cd22 and anti-cd79b antibody drug conjugates are active in different molecular diffuse large b-cell lymphoma subtypes. Leukemia.

[18] Wang P., Hristopoulos M., Clark R., Chen Y., Ellerman D., Mathieu M., Spiess C., Li J., Chalouni C., Sukumaran S. (2017). T cell-dependent bispecific antibody anti-cd79b/cd3 as a potential therapy for b-cell malignancies. Cancer Res..

[19] Spiess C., Merchant M., Huang A., Zheng Z., Yang N.Y., Peng J., Ellerman D., Shatz W., Reilly D., Yansura D.G. (2013). Bispecific antibodies with natural architecture produced by co-culture of bacteria expressing two distinct half-antibodies. Nat. Biotechnol..

[20] Ridgway J.B., Presta L.G., Carter P. (1996). ‘Knobs-into-holes’ engineering of antibody ch3 domains for heavy chain heterodimerization. Protein Eng..

[21] Atwell S., Ridgway J.B., Wells J.A., Carter P. (1997). Stable heterodimers from remodeling the domain interface of a homodimer using a phage display library. J. Mol. Biol..

[22] van Steeg T.J., Bergmann K.R., Dimasi N., Sachsenmeier K.F., Agoram B. (2016). The application of mathematical modelling to the design of bispecific monoclonal antibodies. mAbs.

[23] Rhoden J.J., Dyas G.L., Wroblewski V.J. (2016). A modeling and experimental investigation of the effects of antigen density, binding affinity, and antigen expression ratio on bispecific antibody binding to cell surface targets. J. Biol. Chem..

[24] Leong S.R., Sukumaran S., Hristopoulos M., Totpal K., Stainton S., Lu E., Wong A., Tam L., Newman R., Vuillemenot B.R. (2017). An anti-cd3/anti-cll-1 bispecific antibody for the treatment of acute myeloid leukemia. Blood.

[25] Staflin K., Zuch de Zafra C.L., Schutt L.K., Clark V., Zhong F., Hristopoulos M., Clark R., Li J., Mathieu M., Chen X. (2020). Target arm affinities determine preclinical efficacy and safety of anti-her2/cd3 bispecific antibody. JCI Insight.

[26] Paborsky L.R., Fendly B.M., Fisher K.L., Lawn R.M., Marks B.J., McCray G., Tate K.M., Vehar G.A., Gorman C.M. (1990). Mammalian cell transient expression of tissue factor for the production of antigen. Protein Eng..

[27] Chen X., Dennis M.S., Ebens A.J., Junttila T.T., Kelley R.F., Mathieu M.A., Sun L.L. (2015). Anti-cd3 Antibodies and Methods of Use. International Patent Application Publication.

[28] Ueda O., Wada N.A., Kinoshita Y., Hino H., Kakefuda M., Ito T., Fujii E., Noguchi M., Sato K., Morita M. (2017). Entire cd3ε, δ, and γ humanized mouse to evaluate human cd3-mediated therapeutics. Sci. Rep..

[29] Bello R., Feito M.J., Ojeda G., Portolés P., Rojo J.M. (2009). N-terminal negatively charged residues in cd3varepsilon chains as a phylogenetically conserved trait potentially yielding isoforms with different isoelectric points: Analysis of human cd3varepsilon chains. Immunol. Lett..

[30] Zheng B., Fuji R.N., Elkins K., Yu S.F., Fuh F.K., Chuh J., Tan C., Hongo J.A., Raab H., Kozak K.R. (2009). In vivo effects of targeting cd79b with antibodies and antibody-drug conjugates. Mol. Cancer Ther..

[31] Carrasco-Triguero M., Davis H., Zhu Y., Coleman D., Nazzal D., Vu P., Kaur S. (2016). Application of a plug-and-play immunogenicity assay in cynomolgus monkey serum for adcs at early stages of drug development. J. Immunol. Res..

[32] Cohen S., Chung S., Spiess C., Lundin V., Stefanich E., Laing S.T., Clark V., Brumm J., Zhou Y., Huang C. (2021). An integrated approach for characterizing immunogenic responses toward a bispecific antibody. mAbs.

[33] Peng K., Siradze K., Fischer S.K. (2021). Characterization of robust immune responses to a bispecific antibody, a novel class of antibody therapeutics. Bioanalysis.

[34] Mager D.E., Jusko W.J. (2001). General pharmacokinetic model for drugs exhibiting target-mediated drug disposition. J. Pharmacokinet. Pharmacodyn..

[35] Mager D.E., Krzyzanski W. (2005). Quasi-equilibrium pharmacokinetic model for drugs exhibiting target-mediated drug disposition. Pharm. Res..

[36] Jiang X., Chen X., Carpenter T.J., Wang J., Zhou R., Davis H.M., Heald D.L., Wang W. (2018). Development of a target cell-biologics-effector cell (tbe) complex-based cell killing model to characterize target cell depletion by t cell redirecting bispecific agents. mAbs.

[37] Pan S., Yu H., Surti A., Cheng I., Marks S.D., Brogan P.A., Eleftheriou D., Standing J.F. (2019). Pharmacodynamics of rituximab on b lymphocytes in paediatric patients with autoimmune diseases. Br. J. Clin. Pharmacol..

[38] Wang B., Liang M., Yao Z., Vainshtein I., Lee R., Schneider A., Zusmanovich M., Jin F., O’Connor K., Donato-Weinstein B. (2013). Pharmacokinetic and pharmacodynamic comparability study of moxetumomab pasudotox, an immunotoxin targeting cd22, in cynomolgus monkeys. J. Pharm. Sci..

[39] Li D., Lee D., Dere R.C., Zheng B., Yu S.F., Fuh F.K., Kozak K.R., Chung S., Bumbaca Yadav D., Nazzal D. (2019). Evaluation and use of an anti-cynomolgus monkey cd79b surrogate antibody-drug conjugate to enable clinical development of polatuzumab vedotin. Br. J. Pharmacol..

[40] Ovacik A.M., Li J., Lemper M., Danilenko D., Stagg N., Mathieu M., Ellerman D., Gupta V., Kalia N., Nguy T. (2019). Single cell-produced and in vitro-assembled anti-fcrh5/cd3 t-cell dependent bispecific antibodies have similar in vitro and in vivo properties. mAbs.

[41] Diehl K.H., Hull R., Morton D., Pfister R., Rabemampianina Y., Smith D., Vidal J.M., van de Vorstenbosch C. (2001). A good practice guide to the administration of substances and removal of blood, including routes and volumes. J. Appl. Toxicol..

[42] Hötzel I., Theil F.P., Bernstein L.J., Prabhu S., Deng R., Quintana L., Lutman J., Sibia R., Chan P., Bumbaca D. (2012). A strategy for risk mitigation of antibodies with fast clearance. mAbs.

[43] Ryan A.M., Sokolowski S.A., Ng C.K., Shirai N., Collinge M., Shen A.C., Arrington J., Radi Z., Cummings T.R., Ploch S.A. (2014). Comparative nonclinical assessments of the proposed biosimilar pf-05280586 and rituximab (mabthera^®^). Toxicol. Pathol..

[44] Song L., Xue J., Zhang J., Li S., Liu D., Zhou T. (2021). Mechanistic prediction of first-in-human dose for bispecific cd3/epcam t-cell engager antibody m701, using an integrated pk/pd modeling method. Eur. J. Pharm. Sci. Off. J. Eur. Fed. Pharm. Sci..

[45] Ferl G.Z., Reyes A., Sun L.L., Cheu M., Oldendorp A., Ramanujan S., Stefanich E.G. (2018). A preclinical population pharmacokinetic model for anti-cd20/cd3 t-cell-dependent bispecific antibodies. Clin. Transl. Sci..

[46] Treacy G. (2000). Using an analogous monoclonal antibody to evaluate the reproductive and chronic toxicity potential for a humanized anti-tnfalpha monoclonal antibody. Hum. Exp. Toxicol..

[47] Green J.D., Terrell T.G. (1992). Utilization of homologous proteins to evaluate the safety of recombinant human proteins--case study: Recombinant human interferon-gamma (rhifn-gamma). Toxicol. Lett..

[48] Clarke J., Leach W., Pippig S., Joshi A., Wu B., House R., Beyer J. (2004). Evaluation of a surrogate antibody for preclinical safety testing of an anti-cd11a monoclonal antibody. Regul. Toxicol. Pharmacol..

[49] Bussiere J.L., Martin P., Horner M., Couch J., Flaherty M., Andrews L., Beyer J., Horvath C. (2009). Alternative strategies for toxicity testing of species-specific biopharmaceuticals. Int. J. Toxicol..

[50] Bornstein G.G., Klakamp S.L., Andrews L., Boyle W.J., Tabrizi M. (2009). Surrogate approaches in development of monoclonal antibodies. Drug Discov. Today.

[51] Bumbaca Yadav D., Sharma V.K., Boswell C.A., Hotzel I., Tesar D., Shang Y., Ying Y., Fischer S.K., Grogan J.L., Chiang E.Y. (2015). Evaluating the use of antibody variable region (fv) charge as a risk assessment tool for predicting typical cynomolgus monkey pharmacokinetics. J. Biol. Chem..

[52] Li J., Stagg N.J., Johnston J., Harris M.J., Menzies S.A., DiCara D., Clark V., Hristopoulos M., Cook R., Slaga D. (2017). Membrane-proximal epitope facilitates efficient t cell synapse formation by anti-fcrh5/cd3 and is a requirement for myeloma cell killing. Cancer Cell.

[53] Dostalek M., Gardner I., Gurbaxani B.M., Rose R.H., Chetty M. (2013). Pharmacokinetics, pharmacodynamics and physiologically-based pharmacokinetic modelling of monoclonal antibodies. Clin. Pharmacokinet..

[54] Moore P.A., Zhang W., Rainey G.J., Burke S., Li H., Huang L., Gorlatov S., Veri M.C., Aggarwal S., Yang Y. (2011). Application of dual affinity retargeting molecules to achieve optimal redirected t-cell killing of b-cell lymphoma. Blood.

[55] Carter R.H. (2006). B cells in health and disease. Mayo Clin. Proc..

