# Supplementary material for: Nonclinical Pharmacokinetics and Pharmacodynamics Characterization of Anti-CD79b/CD3 T Cell-Dependent Bispecific Antibody Using a Surrogate Molecule: A Potential Therapeutic Agent for B Cell Malignancies
Source: Pharmaceutics. 2022 Apr 30;14(5):970. doi: 10.3390/pharmaceutics14050970 (PMC9147001; doi:10.3390/pharmaceutics14050970)
Supplement: Supplementary file 1 [file pharmaceutics-14-00970-s001.zip › pharmaceutics-1669051-supplementary.pdf]

pharmaceutics MDPI

Supplementary Materials: Nonclinical Pharmacokinetics and Pharmacodynamics Characterization of Anti-CD79b/CD3 T Cell-Dependent Bispecific Antibody Using a Surrogate Molecule: A Potential Therapeutic Agent for B Cell Malignancies

Rajbharan Yadav, Siddharth Sukumaran, Tanja S. Zabka, Jinze Li, Amy Oldendorp, Gary Morrow, Arthur Reyes, Melissa Cheu, Jessica Li, Jeffrey J. Wallin, Siao Tsai, Laura Sun, Peiyin Wang, Diego Ellerman, Christoph Spiess, Andy Polson, Eric G. Stefanich, Amrita V. Kamath and Meric A. Ovacik

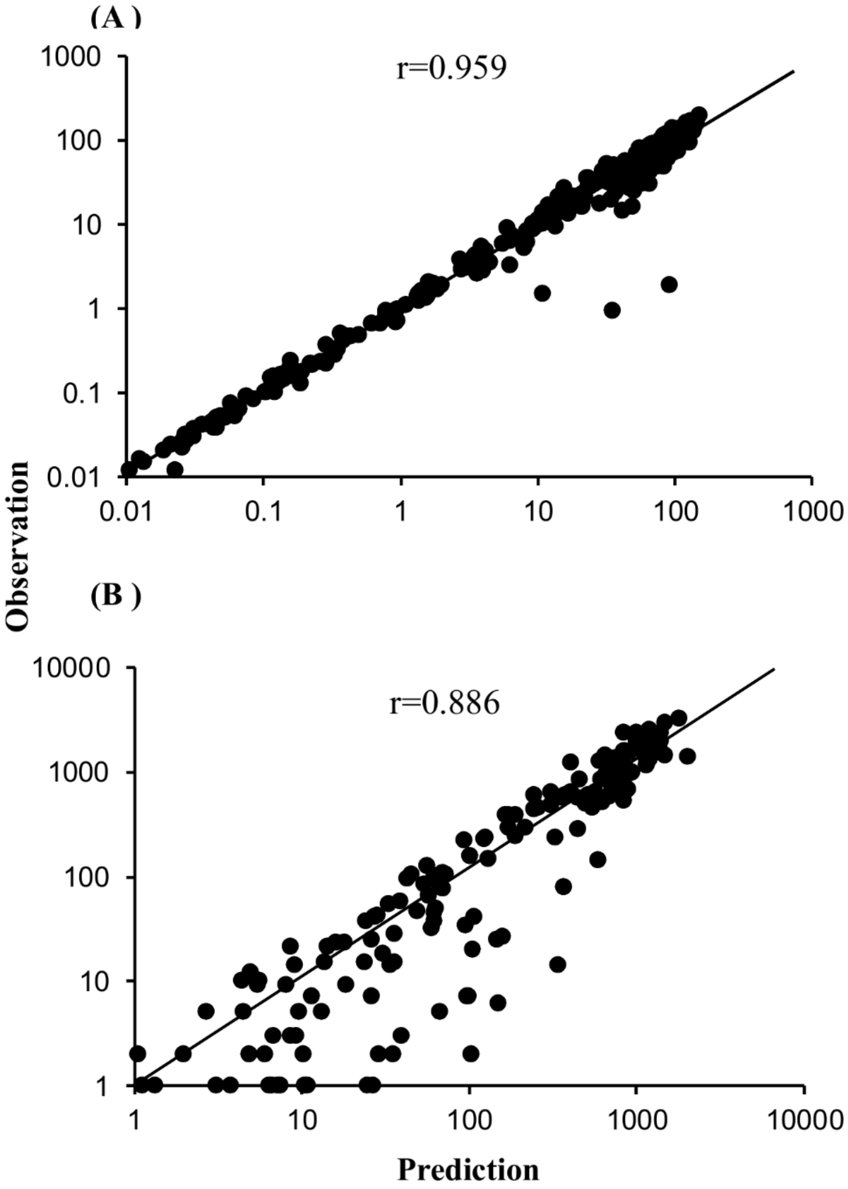

Figure S1. Observed versus predicted serum concentration (A) and B cell (CD20+ B lymphocyte) counts (B) following anti-CD79b/CD3 treatment in cynomolgus monkeys.

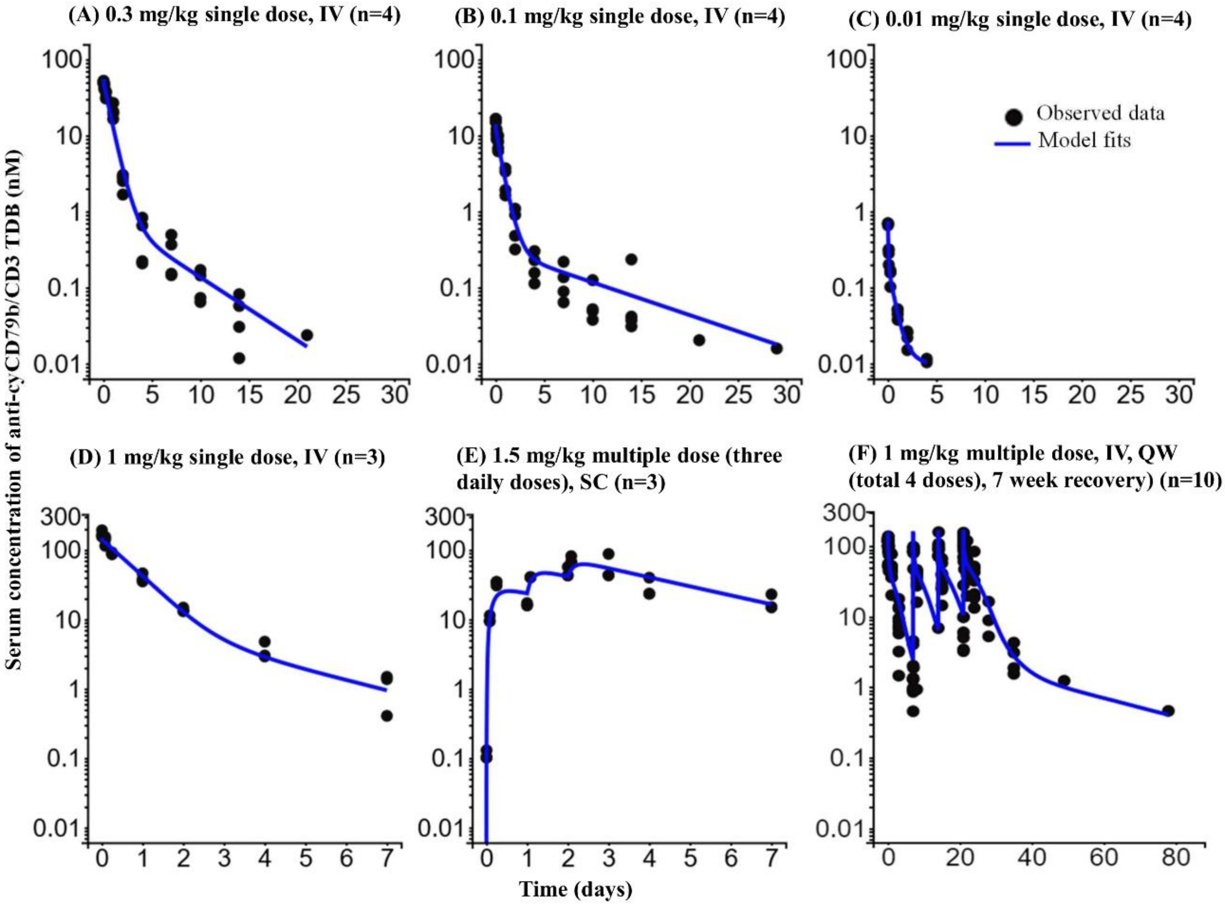

Figure S2. Population PK fits in cynomolgus monkeys receiving surrogate (anti-cyCD79b/CD3) TDB. 0.3 mg/kg IV PK profiles (A), 0.1 mg/kg IV PK profiles (B), 0.01 mg/kg IV PK profiles (C), 1 mg/kg IV PK profiles (D), 1.5 mg/kg, SC daily (total 3 doses) PK profiles (E) and 1 mg/kg IV, QW (total 4 doses with 7-week recovery) PK profiles (F). Circles represent the observed data. Solid blue lines are the population model predictions.

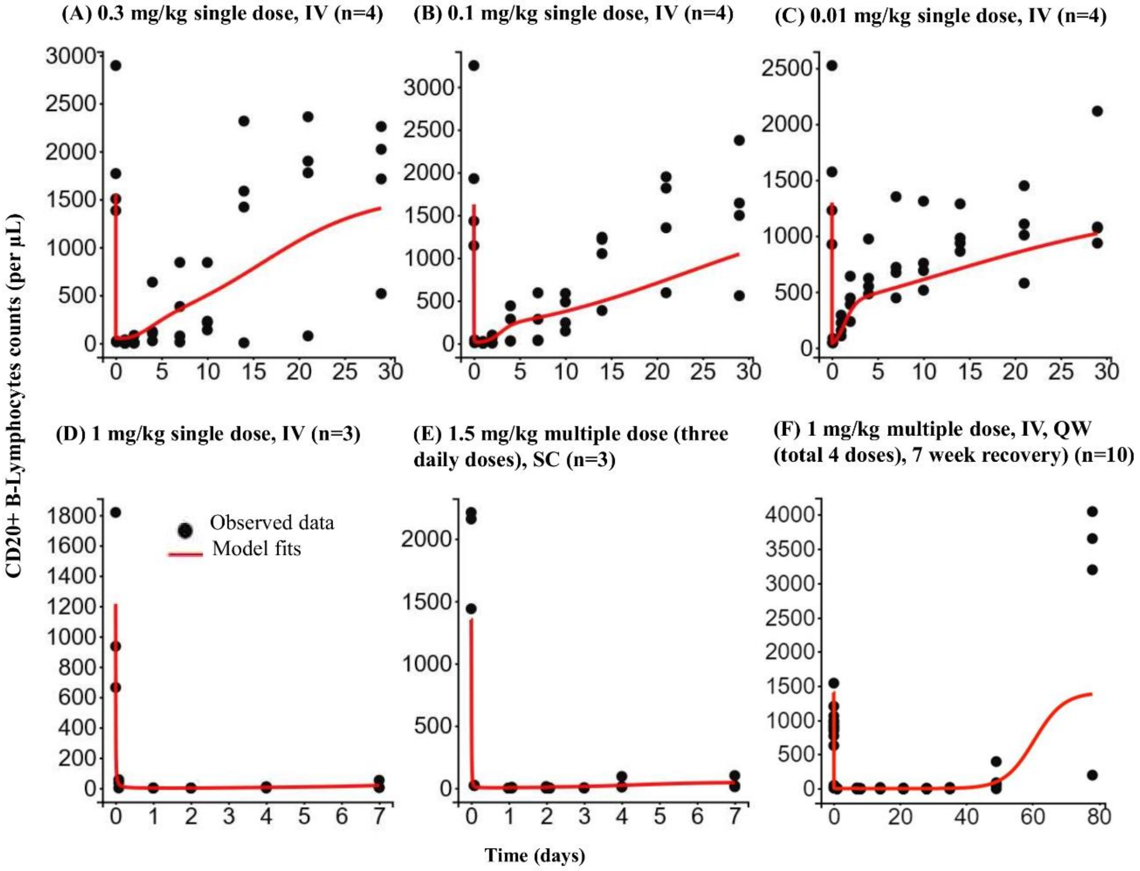

Figure S3. Population PD (B cell counts) fits in cynomolgus monkeys receiving surrogate (anti-cyCD79b/CD3) TDB. 0.3 mg/kg IV PD profiles (A), 0.1 mg/kg IV PD profiles (B), 0.01 mg/kg IV PD profiles (C), 1 mg/kg IV PD profiles (D), 1.5 mg/kg, SC daily (total 3 doses) PD profiles (E) and 1 mg/kg IV, QW (total 4 doses with 7-week recovery) PD profiles (F) doses. Circles represent the observed data. Solid red lines are the population model predictions.

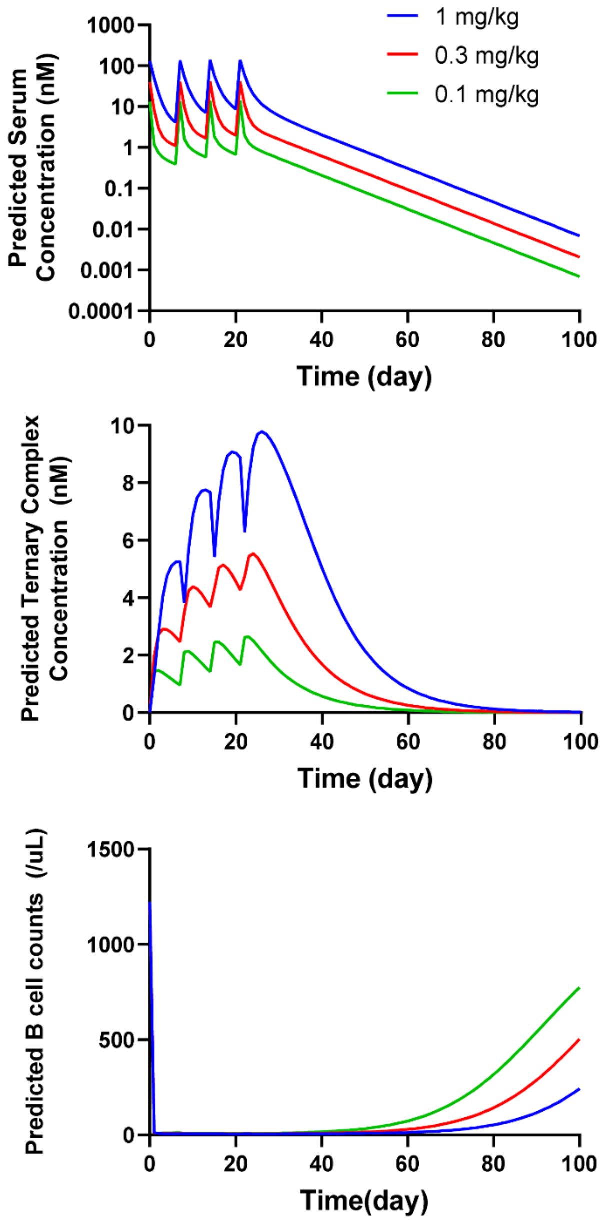

Figure S4. Model predicted anti-cyCD79b/CD3 TDB serum concentration, ternary complex concentration and the B cell depletion following 0.1 mg/kg, 0.3 mg/kg, and 1 mg/kg IV, QW (4 doses). The model predicts a delayed buildup of ternary complex concentrations for increasing doses. The predicted ternary complex concentrations are substantially lower compared to very high serum concentrations at respective doses, however sufficient to drive B cell depletion.

Table S1. Anti-drug antibody (ADA) titers against anti-CD79b/ CD3, anti-gD/CD3-low affinity, Anti-gD/CD3-high affinity TDBs.

Group	Treatment	Subject	Pre-Dose	PK Day 14	PK Day 21	PK Day 28	PK Day 34
1	Anti-CD79b/CD3; 1 mg/kg; IV	1001	<1.70	4.50	5.00	4.97	4.71
		1002	<1.70	4.18	4.97	4.91	4.80
		1003	1.82	4.77	>5.04	4.97	4.90
2	Anti-gD/ CD3-low affinity 1.0 mg/kg, IV	2001	<1.70	<1.70	<1.70	<1.70	<1.70
		2002	<1.70	<1.70	<1.70	<1.70	<1.70
		2003	<1.70	<1.70	3.10	3.96	4.17
3	Anti-gD/CD3-high affinity 1.0 mg/kg, IV	3001	<1.70	4.56	>5.04	5.03	4.73
		3002	<1.70	4.56	>5.04	>5.04	>5.04
		3003	<1.70	3.78	4.91	4.96	4.96

Table S2. Anti-drug antibody (ADA) titers against anti-cyCD79b/ CD3 TDB.

Group	Treatment	Subject	Pre-Dose	TK Day 7	TK Day 14	TK Day 28	TK Day 49	TK Day 78
1	Control (Male)	1001	-	-	<1.30	1.32		
		1002	-	-	-	-		
		1003	-	-	-	-		
		1004	-	-	-	<1.30	-	-
		1005	-	-	<1.30	-	-	-
	Control (Female)	1501	-	-	-	-		
		1502	-	-	-	-		
		1503	-	-	-	-		
		1504	-	-	-	-	<1.30	<1.30
		1505	-	-	-	-	-	-
2	Anti-cyCD79b/CD3 TDB 1.0 mg/kg, IV, QW, (Male)	2001	-	-	2.55	2.74		
		2002	-	-	-	-		
		2003	-	-	2.44	2.99		
		2004	-	-	2.10	3.24	3.00	3.40
		2105	-	-	2.77	-	<1.30	3.74
	Anti-cyCD79b/CD3 TDB 1.0 mg/kg, IV, QW, (Male)	2501	-	-	2.42	4.23		
		2502	-	-	2.49	2.66		
		2503	-	-	1.58	<1.30		
		2505	-	-	1.76	1.82	<1.30	3.57
		2604	-	-	2.21	-	-	-

Dash (-) symbol represent ADA negative samples; minimum reportable titer = 1.30.

Anti- Drug antibody (ADA) Bioanalysis method: The ADA ELISA format utilized anti-CD79b/CD3 or anti-cyCD79b/CD3 or anti-gD/CD3, or anti-gD/CD3 TDB and biotinylated mouse anti-monkey IgG and Streptavidin (SA)-horseradish peroxidase (HRP) as the detection reagents (Cat# 200–032-156, Jackson ImmunoResearch, West Grove, PA). Study samples and negative control pool serum samples were diluted 50-fold for screening. The optical density (OD) signal for each sample was compared with the average signal (n=4) from the negative serum pool. Samples with a mean signal (measured in absorbance units) equal to or greater than the cutpoint were considered positive. Titer values were calculated from serial dilutions of positive samples using the following formula:

$$\text{Titer} = \log\{[(a - c) \div (a - b) \times (e - d)] + d\}$$

Where:

a = the signal of the positive sample or control above the cutpoint

b = the signal of the positive sample or control below the cutpoint

c = the signal of the cutpoint

d = the dilution of the positive sample or control signal above the cutpoint

e = the dilution of the positive sample or control signal below the cutpoint

The cutpoint for each assay was obtained by multiplying the mean optical density of the negative control by the assay cutpoint multiplication factor. The cutpoint multiplication factor was determined using a panel of 30 individual drug-naïve cynomolgus monkey sera, with an estimated false-positive rate of approximately 5% (1–2 samples tested above the assay cutpoint). The panel of individual sera was made of samples obtained from Bioreclamation (Hicksville, NY). Pooled cynomolgus monkey serum was used as the negative control for the assay. An affinity purified cynomolgus antibody was used as positive control
